# Nine minutes is enough: An accelerated fMRI protocol for reliable connectivity mapping in healthy young adults

**DOI:** 10.1080/19585969.2026.2710912

**Published:** 2026-10-14

**Authors:** Adrian Falkowski, Krzysztof Szwed

**Affiliations:** a Faculty of Mathematics and Computer Science, Nicolaus Copernicus University, Toruń, Poland; b Institute of Advanced Studies, Nicolaus Copernicus University, Toruń, Poland; c Faculty of Medicine, Bydgoszcz University of Science and Technology, Bydgoszcz, Poland

**Keywords:** fMRI, Multiband, brain connectivity, graph theory, Resting-State, topological stability

## Abstract

**Introduction:**

Resting‑state fMRI is widely used to map brain connectivity, yet the field lacks a clear, data‑driven standard for acquisition duration. We compared traditional Single‑Band (SB) imaging with an accelerated Multiband factor 4 (MB4) protocol to identify a time‑efficient approach for capturing stable network topology.

**Methods:**

Twenty‑five healthy young adults completed two sessions one month apart, each including counterbalanced 13‑minute SB and MB4 scans. Test–retest reliability was evaluated across multiple durations using Portrait Divergence, a multiscale graph‑theoretic metric. Supplementary analyses compared Portrait Divergence with the field-standard Intraclass Correlation Coefficient, assessed robustness across preprocessing pipelines and parcellation schemes, and examined MB4’s ability to capture both cortical and subcortical connectivity.

**Results:**

MB4 consistently outperformed SB across all tested durations, with peak topological stability at 9 minutes. Stability declined beyond this point, likely due to cumulative motion artefacts. Supplementary analyses confirmed MB4’s robustness across preprocessing strategies and atlas choices and demonstrated effective whole‑brain coverage.

**Conclusions:**

In healthy young adults, MB4 captures network topology more effectively than SB and reaches peak stability at 9 minutes before declining, challenging the assumption that longer scans are always better. These findings require validation in clinical, paediatric, and geriatric cohorts.

## Introduction

Resting-state functional magnetic resonance imaging (rs-fMRI) is widely used to map brain connectivity, yet the field still lacks consensus on the optimal scan duration. Shorter acquisitions improve participant comfort, reduce motion-related artefacts, and streamline research workflows, but they may fail to preserve reliable connectivity estimates. Multiband imaging offers a powerful strategy for accelerating data collection through simultaneous slice acquisition (Feinberg et al. 2010; Moeller et al. 2010; Xu et al. 2013), although the trade-off between its increased sampling speed and test–retest reliability remains insufficiently understood. Identifying the shortest multiband scan duration that still yields robust and reproducible connectivity estimates is therefore essential for establishing time-efficient, standardised rs-fMRI protocols.

Previous work has established multiband factor 4 (MB4) as the most efficient setting for rs-fMRI (Risk et al. 2021) and shown that pairing it with factor-2 in-plane acceleration preserves SNR and functional connectivity (Cahart et al. 2023). Guided by this evidence, we selected the MB4 configuration to compare it directly with traditional single-band (SB) imaging and determine the scan duration required to obtain reliable connectivity estimates.

Despite extensive efforts to optimise rs-fMRI parameters, inconsistent analytical approaches continue to hinder standardisation (Smitha et al. 2017). Common reliability metrics such as intra-class correlation (ICC) and connectome fingerprinting emphasise individual edges (Graff et al. 2022), making them vulnerable to noisy, low-reliability connections and limiting their sensitivity to distributed, system-level network changes (Luppi et al. 2024). To address this limitation, we used Portrait Divergence (Bagrow and Bollt 2019), an information-theoretic metric that compares whole-brain connectivity graphs across multiple topological scales. It constructs ‘network portraits’, converts them into probability distributions, and quantifies their similarity using Jensen–Shannon divergence (Lin 1991). Although not yet clinically interpretable, Portrait Divergence is well suited for detecting subtle, system-wide reconfigurations that edge-wise metrics often overlook, underscoring its relevance for future psychiatric and neurological research.

In this study, we use Portrait Divergence to compare the performance of MB4 and SB in providing stable whole-brain connectivity estimates and to identify the shortest scan duration that achieves reliable topological stability. We benchmark these results against the field-standard ICC and test robustness across preprocessing pipelines and parcellation schemes to ensure generalisability. We hypothesised that MB4’s superior sampling efficiency would result in greater topological stability compared to SB, with peak performance emerging at an intermediate scan duration.

## Methods

### Study design, setting and participants

This prospective cohort study, approved by the local bioethics committee (KB 81/2023) and conducted in accordance with the Declaration of Helsinki, recruited 25 healthy university students (13 females, age 23.98 +/- 1.16) who had no contraindications to MRI and provided written informed consent. Participants underwent two scanning sessions, one month apart. Each session included 13-minute SB and MB4 sequences, counterbalanced within and between sessions to address possible confounding habituation effects during analysis, and participants remained blinded to which sequence was acquired first. During scanning, participants were instructed to fixate on a central crosshair, stay awake, and refrain from focused thought.

### Data acquisition and preprocessing

Data were acquired using a 3 T scanner (Philips, Ingenia, Philips Medical Systems) equipped with a 32-channel head coil. T1-weighted structural images were obtained with a gradient echo sequence (3D turbo field echo; 181 contiguous sagittal 1.0 mm slices; field of view = 187 mm x 267 mm x 262 mm; time to repetition = 8.1 ms; time to echo = 3.7 ms; flip angle = 8°; isotropic 1 mm voxel). For comparing the SB and MB4 sequences, we designed their acquisition parameters to be as similar as practically feasible, with details presented in Table 1.

**Table 1.** fMRI Sequence Parameters: Single-Band vs. Multiband.

Parameter	Single-Band	Multiband
Multiband Factor	1	4
In-Plain Acceleration	2	2
Repetition Time (s)	3	0.69
No of Volumes	256	1122
Echo Time (s)	0.035	0.03
Echo Spacing (s)	0.0001	0.0003
Number of Slices	44	44
Slice Thickness (mm)	3	3
Gap (mm)	0.5	0.5
In-plane Resolution (mm x mm)	1.736 x 1.736	1.953 x 1.953
Field of View (mm x mm x mm)	254 x 285 x 225	254 x 285 x 225
Flip Angle (º)	90	52
Phase Encoding Direction	anterior-posterior	anterior-posterior

Comparison of Single-Band and Multiband fMRI Sequence Parameters.

To ensure a fair comparison of SB and MB4 acquisitions across varying time points, we applied identical preprocessing to both sequences. We used fMRIPrep version 25.0.0 (Esteban et al. 2019), a robust and widely adopted tool for standardised preprocessing and high reproducibility; a detailed methods description is provided in (Supplementary Material Section 1: Preprocessing of MRI Data). Crucially, fMRIPrep registers all functional data to the MNI152NLin2009cAsym template, a standard brain space with 2 mm isotropic voxels. This process ensures that the SB and MB4 data are aligned to the exact same anatomical reference space, enabling direct, voxel-wise comparisons of brain activity between the two acquisition types. To investigate the effect of scan duration, we then partitioned the 13-minute SB and MB scans into shorter segments, ranging from 5 to 13 min in 1-minute increments. Subsequent preprocessing was conducted using AFNI’s 3dTproject (Cox 1996), which included stringent motion artefact mitigation *via* censoring based on framewise displacement exceeding 0.4 mm. This threshold was selected according to recent recommendations indicating an optimal balance between preserving sufficient data volumes and minimising motion-related artefacts in functional connectivity metrics (Birn 2023). To mitigate the influence of confounding signals, nuisance regression was performed, including six head motion parameters (three translations, three rotations) along with aCompCor (Behzadi et al. 2007) derived signals from cerebrospinal fluid and white matter. Bandpass filtering was applied within the frequency range of 0.008–0.09 Hz. Degrees of freedom were obtained directly from AFNI’s 3dTproject output, which performs censoring, nuisance regression, and temporal filtering within a single linear regression framework. The reported degrees of freedom correspond to the effective degrees of freedom of the full model, reflecting the number of independent observations remaining after accounting for all modelled confounds. Quality control criteria derived from (Birn 2023) were subsequently applied, excluding subjects with fewer than 15 degrees of freedom remaining after preprocessing or those retaining less than 240 s of usable data post-censoring. Supplementary Material (see Section 10: Data Availability Across Trimming Durations and Motion Censoring) provides a detailed summary of the number of volumes and usable data for both MB4 and SB sequences.

### Data processing and statistical analysis

In this study, we employed a brain map combining two established functional atlases: Schaefer 400 (Schaefer et al. 2018) (cortical) and Tian S1 for 3 T MRI (Tian et al. 2020) (subcortical). This integrated approach was adopted to promote a more comprehensive understanding of brain connectivity by capturing interactions between both cortical and subcortical areas. To ensure robust data quality, we excluded regions of interest (ROIs) exhibiting less than 80% brain signal coverage, resulting in a final set of 355 ROIs. Functional connectivity was then assessed by computing Fisher’s z-transformed Pearson correlations between ROI time series. Negative correlations were set to zero, yielding a 355x355 connectivity matrix for each participant. Network representations were subsequently constructed using weighted edges, followed by proportional edge filtering at a threshold of 20%, as recommended by Luppi et al. (2024).

Statistical analyses were performed using linear mixed-effects models to examine the effects of acquisition method (MB4 vs. SB) and scan duration (5–13 min) on Portrait Divergence. In the full model, both factors were included as fixed effects, while individual differences were accounted for by modelling subject as a random effect. Additionally, separate models were estimated within each acquisition method to assess the influence of scan duration independently. To assess the significance of main effects, F-tests based on type II ANOVA were applied.

To explore potential nonlinear effects of scan duration on Portrait Divergence, quadratic regression models were fit separately for the SB and MB4 acquisition methods. These models were used to estimate the overall shape of the relationship and, critically, to identify the scan duration associated with the lowest (best) divergence value. The robustness and statistical significance of these patterns were assessed using standard regression diagnostics: adjusted R^2^ and F-tests.

Most statistical procedures were implemented using R (accessed *via* rpy2 from Python). Specifically, mixed-effects models were fit using the lme4 (Bates et al. 2015) and lmerTest (Kuznetsova et al. 2017) packages, and ANOVA was conducted using car (Fox and Weisberg 2019). Nonlinear curve fitting was performed in Python using scipy.optimise.curve_fit (Virtanen et al. 2020). Portrait Divergence was computed using the Python implementation provided by the original authors (Bagrow and Bollt 2019), available at https://github.com/bagrow/network-portrait-divergence.

## Main results

All twenty-five participants completed two fMRI sessions each. However, processing issues rendered three MB4 acquisitions unusable due to objective DICOM-to-NIfTI conversion failures, resulting in 25 SB scan sets (two per participant) and 22 MB4 scan sets (two per participant, excluding three compromised acquisitions) for final analysis.

Figure 1 and Table 2 present a comprehensive analysis of Portrait Divergence for MB4 and SB acquisitions across different scan lengths. While MB4 consistently outperformed SB, exhibiting the lowest (best) Portrait Divergence around 9 min, longer durations did not yield further improvements.

**Figure 1.**
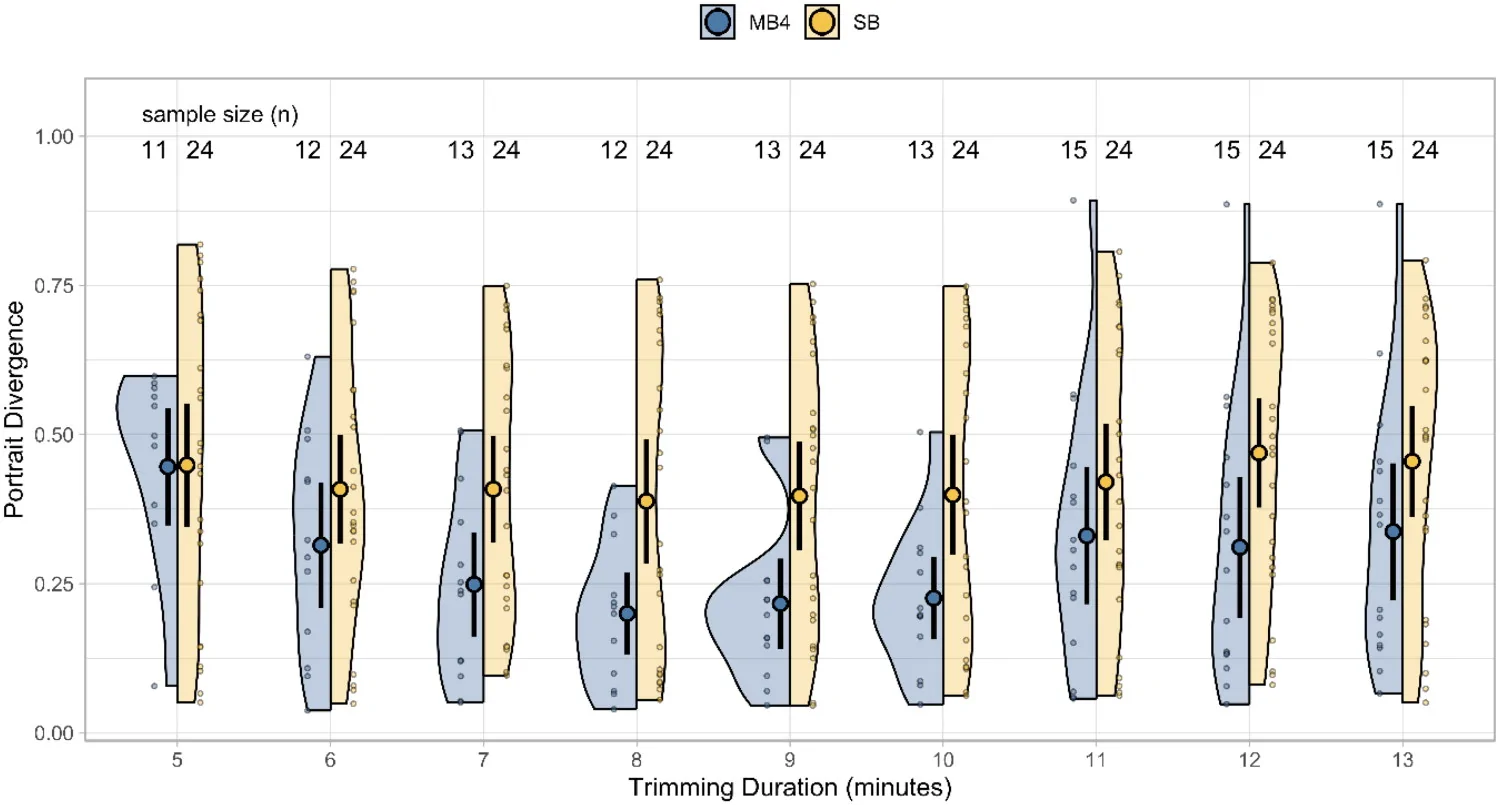
Topological stability (portrait divergence) by method and scan duration. Split violin plot depicting the distribution of Portrait Divergence values across different trimmed scan durations (in minutes) for Multiband factor 4 (MB4) and Single-band (SB) acquisition methods. Violin shapes illustrate the kernel density estimates for each method, with jittered dots representing individual observations. Black point-ranges indicate mean values with 95% confidence intervals. Sample sizes (n) reported at the top of the plot indicate the number of subjects retained after post-censoring quality control for each duration and acquisition method. The resulting variation reflects the strict quality-control criteria applied in the main analysis. Importantly, the same pattern is reproduced under more permissive motion thresholds, yielding balanced-cohort analyses. This demonstrates that the observed findings are robust to specific data-quality constraints and the associated sample-size differences (see Supplementary Material, Section 5: Stability Analyses under Parcellation Scheme and Data-Quality Constraints).

**Table 2.** Statistical Summary: MB4 vs. SB and Optimal Scan Duration.

Analysis	Result
**Comparison of MB4 vs SB methods:** Mean difference:	−0.128, 95% CI: −0.172, −0.083 ANOVA *p* < 0.001***
**Effect of scan length:**	
- within SB:	ANOVA *p* = 1.000
- within MB4:	ANOVA *p* = 0.002**
**Quadratic model fit:**	
- SB: optimum at	8.45 min, R²_adj = 0.692, *p* = 0.036*
- MB4: optimum at	9.20 min, R²_adj = 0.659, *p* = 0.049*
**Equations of fitted quadratic curves:**	
- SB:	*y* = 0.0037t² + −0.0628t + 0.6608
- MB4:	*y* = 0.0111t² + −0.2033t + 1.1519

Summary of statistical analyses: comparison between Multiband factor 4 (MB4) and singleband (SB) acquisition methods, and quadratic fits across scan lengths. All p-values were corrected for multiple comparisons using the Bonferroni method. Significance levels after correction: *p < 0.05, **p < 0.01, ***p < 0.001.

## Supplementary results and additional methodological considerations

### ICC Benchmarking Analyses

To benchmark Portrait Divergence against a field-standard measure, we performed an edge-wise ICC analysis (see Supplementary Material, Section 2: ICC Benchmarking Analyses). ICC values were calculated for each functional connectivity edge using a two-way mixed-effects model, single-measurement, consistency definition [ICC(3,1)], the commonly applied setting for test–retest stability in functional connectivity studies (Noble et al. 2019). Test-retest reliability was computed separately for MB4 and SB acquisitions and summarised as global mean ICC values across all tested scan durations (5–13 min). Global mean ICC values stabilised around 0.35–0.40 at longer scan durations, consistent with prior methodological reports (Birn, 2013). However, this plateau diverged from our main findings, where MB4 demonstrated clear superiority and an optimal topological stability peak at 9 min. This discrepancy highlights the key advantage of Portrait Divergence: whereas ICC averages the test-retest reliability of individual connections and is diluted by many low-reliability edges, Portrait Divergence measures the consistency of the entire network’s topology across scales. Supporting this, ΔICC maps, which reflected differences in test–retest reliability between the MB4 and SB acquisitions, revealed localised increases in stability for MB4 within key nodes—most notably in the Default Mode Network—that were statistically overshadowed in the global ICC mean. These subtle but structurally important effects are precisely what Portrait Divergence is designed to capture.

### Model diagnostics

To ensure that the mixed effects models provided an adequate fit to the data, we conducted a set of standard model diagnostic evaluations (see Supplementary Material, Section 3: Model Diagnostics). These diagnostics assessed the core assumptions underlying the linear mixed effects framework, including the distributional properties of residuals and random effects, the presence of heteroscedasticity, and potential dependencies between residuals and predicted random effects. Overall, these diagnostics did not indicate major departures from normality, pronounced heteroscedasticity, or other systematic patterns that would call the validity of the model inferences into question.

### Power analysis

A formal post-hoc power analysis was conducted (see Supplementary Material, Section 4: Power Analysis), confirming that the final sample size offered sufficient statistical power to detect the robust effect sizes observed in both the MB4 vs. SB comparison and the duration-dependent stability analyses.

### Robustness of MB4 stability across preprocessing and atlas choices

It is important to note that optimal preprocessing remains a complex and debated topic, as discussed in Graff et al. (2022). Therefore, we prioritised preprocessing steps that are applicable to both SB and MB data, align with recent research recommendations, and are widely used in the field (Birn 2023; Luppi et al. 2024). This approach aimed to provide a direct and unbiased comparison of acquisition stability, rather than focusing on identifying the theoretically optimal preprocessing strategy for each acquisition type. Nevertheless, mindful that overly strict preprocessing parameters could lead to excessive participant exclusion, we conducted a supplementary analysis using a 1 mm censoring threshold (see Supplementary Material, Section 5: Stability Analyses under Parcellation Scheme and Data-Quality Constraints). This threshold was chosen as part of a sensitivity analysis, reflecting a common, less restrictive standard used in methodological literature, allowing us to retain a greater number of degrees of freedom, especially for shorter scan lengths. (Birn 2023) This relaxed criterion excluded only one MB4 acquisition, further confirming MB4’s consistent and statistically significant advantage over SB, even in high-motion data. This robustness provides a practical advantage for researchers leveraging MB4, allowing them to retain motion-prone participants in their cohorts, minimising attrition and improving study feasibility.

Previous research has raised concerns that MB acquisition may exhibit reduced test-retest reliability in subcortical regions compared to SB acquisition (Cahart et al. 2023). To address this, we conducted a supplementary analysis using the Schaefer atlas alone (see Supplementary Material, Section 5: Stability Analyses under Parcellation Scheme and Data-Quality Constraints). Interestingly, raw topological stability values were better in the combined atlas compared to the cortical-only atlas, although the difference was not statistically significant. These findings indicate that integrating subcortical regions does not compromise topological stability, allowing us to confidently employ the more informative dual-atlas approach for rs-fMRI acquisition leveraging MB4.

Finally, to ensure the generalisability of our findings across different parcellation schemes—following the approach of Luppi et al. (2024) we conducted a supplementary analysis assessing topological stability metrics across varying granularities of the functional Schaefer atlas (100 and 200 parcels) (see Supplementary Material, Section 5: Stability Analyses under Parcellation Scheme and Data-Quality Constraints). Across all variations, MB4 acquisition consistently demonstrated a statistically significant advantage, with a robust divergence emerging around the 9-minute mark. This topological stability across different brain atlases further reinforces the benefits of MB4 for rs-fMRI studies.

### Statistical modeling of Motion Accumulation

To directly test whether motion artefacts contributed to the decline in topological stability, we conducted post hoc analyses modelling motion trajectories across scan durations (see Supplementary Material, Section 6: Statistical Modelling of Motion Accumulation). A linear mixed-effects model was applied to estimate how mean framewise displacement evolved over time and whether trajectories differed between sessions. In MB4 acquisitions, motion increased significantly with duration and diverged across sessions, whereas SB trajectories remained stable. Complementary piecewise regression with a fixed breakpoint at 9 min further demonstrated a change in slope for MB4, consistent with the observed peak in Portrait Divergence stability. Together, these analyses indicate that accumulating, session-inconsistent motion after approximately 9 min compromises topological stability in MB4 data, explaining the decline in topological stability beyond this point.

### MB4 Undersampling Analyses

To isolate the effect of MB4’s superior temporal sampling efficiency, we generated a sub-sampled MB4 dataset by retaining only the number of volumes equivalent to those acquired with the SB sequence within the same time window (see Supplementary Material, Section 7: MB4 Undersampling Analyses). The topological stability of this sub-sampled MB4 dataset was then compared against the full MB4 and SB datasets. When deprived of its native sampling advantage, MB4 exhibited a statistically significant loss of stability relative to the full MB4 data. These findings indicate that MB4’s stability benefit is contingent on preserving its higher temporal resolution, underscoring the critical role of acquisition parameters in shaping test–retest outcomes.

### Edge Thresholding Sensitivity Analyses

To assess the robustness of our findings to thresholding choices, we repeated the Portrait Divergence analyses using alternative edge-retention thresholds of 10%, 15%, 25% and 30%, in addition to the primary 20% setting (see Supplementary Material, Section 8: Edge Thresholding Sensitivity Analyses). For each threshold, functional connectivity matrices were reconstructed and Portrait Divergence values recalculated across all scan durations. The resulting stability profiles showed consistent patterns across thresholds, with the 9-minute peak preserved, indicating that the observed effects are not dependent on the specific 20% threshold but reflect broader topological properties of the data.

### Sliding Window Sensitivity analysis

To ensure that our findings were not dependent on the specific temporal segments selected during cumulative trimming, we performed a secondary sensitivity analysis using fixed-length sliding windows (see Supplementary Material, Section 9: Sliding Window Sensitivity Analysis). By shifting these 5–10-minute windows across the entire scan in 1-minute increments and aggregating the resulting Portrait Divergence values, we accounted for temporal variability and potential nested dependencies. This approach allowed us to verify that MB4 maintained lower divergence than SB across all window lengths. Crucially, because sliding windows sample and average across all portions of the scan—including later segments susceptible to motion-related degradation—the contrast between specific durations is inherently reduced. Consequently, this smoothing mathematically constrains the model’s capacity to reach a threshold of statistical significance at a precise timepoint. Nevertheless, quadratic model fits still converged on an optimum at approximately 9 min, providing support for our primary findings.

## Discussion

Our study shows that in healthy young adults MB4 demonstrates superiority in topological stability over SB acquisition, peaking at the 9-minute mark. Although both sequences showed comparable stability at the shortest 5-minute duration, MB4 maintained a consistent statistical advantage across the remainder of the tested range. Crucially, this effect proved robust to variations in preprocessing protocols, atlas resolution, and regional coverage—highlighting MB4’s adaptability for whole-brain graph-theoretical analysis and its benefits for multi-visit designs.

MB4’s superior topological stability is likely driven by its enhanced sampling efficiency. By offering higher temporal resolution within the same time frame, MB4 produces richer datasets and improves statistical power for estimating functional connectivity, even with a modest reduction in signal-to-noise ratio (SNR) (Birn 2023; Barth et al. 2016; Smitha et al. 2018). The 9-minute peak likely reflects an optimal balance between acquiring sufficient data and preventing degradation of topological stability associated with longer scan durations.

The ideal scan length for resting-state fMRI (rs-fMRI) has long been debated, with prior studies reporting plateaus in average connectivity strength around 9–12 min (Birn et al. 2013) and stabilisation of identification accuracy after approximately 5 min (Finn et al. 2015). Recent expert consensus on clinical fMRI applications recommends a minimum scan duration of 6 min, acknowledging that while longer scans enhance functional connectivity test-retest reliability, gains tend to plateau beyond 13 min (Kumar et al. 2024). Building on this work, our analysis demonstrates that the optimal duration is not a fixed value but depends critically on the metric used to assess test-retest reliability. By incorporating a topology-sensitive, multiscale approach, we identified a distinct 9-minute peak in network stability, reflecting the point at which additional data volumes are offset by motion-related degradation. This finding highlights that conventional edge-based or identification metrics, while useful for broad comparisons, may overlook temporally dynamic changes in network organisation. In contrast, topology-sensitive measures capture these subtle shifts, offering a more precise benchmark for protocol optimisation and underscoring the importance of temporal resolution in future connectomics research.

Longer scans introduce important trade-offs. In our supplementary analyses of motion accumulation, head motion in MB4 acquisitions increased systematically with scan duration and diverged across sessions after the 9-minute mark. This accumulation not only elevated the number of censored volumes but also introduced session-specific variability, which can undermine test-retest reliability in longitudinal designs. Even with advanced denoising strategies, excessive motion irreversibly distorts network topology, obscuring genuine connectivity patterns and contributing to the decline in graph-based test-retest reliability observed beyond the optimal window (Balachandrasekaran et al. 2022).

Taken together, these findings emphasise the importance of carefully optimising scan duration—not only to capture stable connectomes but also to minimise artefacts. Based on our results, a 9-minute MB4 acquisition emerges as a time-efficient, topology-sensitive protocol for healthy young adults scanned on 3 T MR systems, offering a strong methodological benchmark while guiding further research to extend these findings to additional populations and settings.

### Limitations

Several limitations should be acknowledged. First, our sample consisted exclusively of healthy university students, which limits the generalisability of our findings to clinical populations or individuals across different age ranges, who often exhibit significantly higher and more unpredictable head motion. Second, while our protocol accounts for standard noise, the potential influence of acceleration-induced artefacts—including interslice leakage (Risk et al. 2018; Xu et al. 2013; McNabb et al. 2020) and other recently described multiband-specific phenomena (Tubiolo et al. 2024)—cannot be fully excluded. Third, we did not collect physiological measures to track vigilance or arousal. Incorporating such measures in future work would help account for fluctuations in wakefulness, which can influence functional connectivity independently of head motion (Tagliazucchi and Laufs 2014). Fourth, all data were acquired using a single MRI scanner; therefore, results may vary across scanners from different manufacturers or with differing hardware configurations. Fifth, while we used traditional Pearson’s correlation matrices to represent functional connectivity, we acknowledge that many alternative approaches exist—such as partial correlations, mutual information, and model based estimators—which capture different aspects of brain organisation. Future research should explore whether the 9-minute MB4 protocol demonstrates similar benefits over SB within these complementary analytical frameworks. Sixth, although the 9-minute MB4 protocol is statistically more reliable than the traditional SB sequence at capturing the brain’s functional networks, the clinical relevance of this advantage remains to be determined.

## Supplementary Material

SBvsMB4_Supplementary_Materials_R3.pdf
