# Supplementary material for: Nine minutes is enough: An accelerated fMRI protocol for reliable connectivity mapping in healthy young adults
Source: Dialogues Clin Neurosci. 2026 Oct 14;29(1):24–31. doi: 10.1080/19585969.2026.2710912 (PMC13670261; doi:10.1080/19585969.2026.2710912)
Supplement: Supplementary file 1 [file TDCN_A_2710912_SM6040.pdf]

1 Preprocessing of MRI Data

Results included in this manuscript come from preprocessing performed using *fMRIPrep* 25.0.0 (Esteban et al. [2019]; Esteban et al. [2018]; RRID:SCR_016216), which is based on *Nipype* 1.9.2 (Gorgolewski et al. [2011]; Gorgolewski et al. [2018]; RRID:SCR_002502).

Preprocessing was performed using a consistent *fMRIPrep* configuration across all subjects in a Singularity environment. Outputs were generated in *MNI152NLin2009cAsym* space at native resolution (`MNI152NLin2009cAsym:res-native`). FreeSurfer surface reconstruction was not performed (`run_reconall = false`), and ICA-AROMA was not used. BOLD-to-anatomical co-registration was performed with six degrees of freedom. Motion outliers were annotated using thresholds of framewise displacement > 0.5 mm and standardized DVARS > 1.5.

Anatomical data preprocessing A total of 2 T1-weighted (T1w) images were found within the input BIDS dataset. Each T1w image was corrected for intensity non-uniformity (INU) with `N4BiasFieldCorrection` [Tustison et al., 2010], distributed with ANTs 2.5.4 [Avants et al., 2008, RRID:SCR_004757]. The T1w-reference was then skull-stripped with a *Nipype* implementation of the `antsBrainExtraction.sh` workflow (from ANTs), using OASIS30ANTs as target template. Brain tissue segmentation of cerebrospinal fluid (CSF), white-matter (WM) and gray-matter (GM) was performed on the brain-extracted T1w using `fast` [FSL (version unknown), RRID:SCR_002823, Zhang et al., 2001]. An anatomical T1w-reference map was computed after registration of 2 <module 'nipype.interfaces.image' from '/opt/conda/envs/fmriprep/lib/python3.12/site-packages/nipype/interfaces/image.py'> images (after INU-correction) using `mri_robust_template` [FreeSurfer 7.3.2, Reuter et al., 2010]. Volume-based spatial normalization to one standard space (MNI152NLin2009cAsym) was performed through nonlinear registration with `antsRegistration` (ANTs 2.5.4), using brain-extracted versions of both T1w reference and the T1w template. The following template was were selected for spatial normalization and accessed with *TemplateFlow* [24.2.2, Ciric et al., 2022]: *ICBM 152 Nonlinear Asymmetrical template version 2009c* [Fonov et al. [2009], RRID:SCR_008796; TemplateFlow ID: MNI152NLin2009cAsym].

Functional data preprocessing For each of the 4 BOLD runs found per subject (across all tasks and sessions), the following preprocessing was performed. First, a reference volume was generated, using a custom methodology of *fMRIPrep*, for use in head motion correction. Head-motion parameters with respect to the BOLD reference (transformation matrices, and six corresponding rotation and translation parameters) are estimated before any spatiotemporal filtering using `mcflirt` [FSL , Jenkinson et al., 2002]. The BOLD reference was then co-registered to the T1w reference using `mri_coreg` (FreeSurfer) followed by `flirt` [FSL , Jenkinson and Smith, 2001] with the boundary-based registration [Greve and Fischl, 2009] cost-function. Co-registration was configured with six degrees of freedom. Several confounding time-series were calculated based on the *preprocessed BOLD*: framewise displacement (FD), DVARS and three region-wise global signals. FD was computed using two formulations following Power (absolute sum of relative motions, Power et al. [2014]) and Jenkinson (relative root mean square displacement between affines, Jenkinson et al. [2002]). FD and DVARS are calculated for each functional run, both using their implementations in *Nipype* [following the definitions by Power et al., 2014]. The three global signals are extracted within the CSF, the WM, and the whole-brain masks. Additionally, a set of physiological regressors were extracted to allow for component-based noise correction [*CompCor*, Behzadi et al., 2007]. Principal components are estimated after high-pass filtering the *preprocessed BOLD* time-series (using a discrete cosine filter with 128s cut-off) for the two *CompCor* variants: temporal (tCompCor) and anatomical (aCompCor). tCompCor components are then calculated from the top 2% variable voxels within the brain mask. For aCompCor, three probabilistic masks (CSF, WM and combined CSF+WM) are generated in anatomical space. The implementation differs from that of Behzadi et al. in that instead of eroding the masks by 2 pixels on BOLD space, a mask of pixels that likely contain a volume fraction of GM is subtracted from the aCompCor masks. This mask is obtained by thresholding the corresponding partial volume map at 0.05, and it ensures components are not extracted from voxels containing a minimal fraction of GM. Finally, these masks are resampled into BOLD space and binarized by thresholding at 0.99 (as in the original implementation). Components are also calculated separately within the WM and CSF masks. For each CompCor decomposition, the k components with the largest singular values are retained, such that the retained components' time series are sufficient to explain 50 percent of variance across the nuisance mask (CSF, WM, combined, or temporal). The remaining components are dropped from consideration. The head-motion estimates calculated in the correction step were also placed within the corresponding confounds file. The confound time series derived from head motion estimates and global

signals were expanded with the inclusion of temporal derivatives and quadratic terms for each [Satterthwaite et al., 2013]. Frames that exceeded a threshold of 0.5 mm FD or 1.5 standardized DVARS were annotated as motion outliers. Additional nuisance timeseries are calculated by means of principal components analysis of the signal found within a thin band (*crown*) of voxels around the edge of the brain, as proposed by [Patriat et al., 2017]. All resamplings can be performed with *a single interpolation step* by composing all the pertinent transformations (i.e. head-motion transform matrices, susceptibility distortion correction when available, and co-registrations to anatomical and output spaces). Gridded (volumetric) resamplings were performed using `nitransforms`, configured with cubic B-spline interpolation.

Many internal operations of *fMRIPrep* use *Nilearn* 0.11.1 [Abraham et al., 2014, RRID:SCR_001362], mostly within the functional processing workflow. For more details of the pipeline, see the section corresponding to workflows in *fMRIPrep*'s documentation.

1.0.1 Copyright Waiver

The above boilerplate text was automatically generated by fMRIPrep with the express intention that users should copy and paste this text into their manuscripts *unchanged*. It is released under the CC0 license.

2 ICC Benchmarking Analyses

We performed complementary reliability analyses based on the Intraclass Correlation Coefficient (ICC), used . For each acquisition type (SB and MB4) and each trimmed scan duration between 5 and 13 minutes, ICC values were computed for every functional connectivity edge using a two-way mixed-effects, single-measurement, consistency model [ICC(3,1)], implemented in R (package `irr` [Gamer et al., 2012]). Test–retest reliability was evaluated across the two sessions, treating them as repeated measurements for the same set of participants.

Edge-wise ICC matrices were first summarized into global indices by averaging over the upper triangle, yielding mean ICC values with corresponding standard deviations for SB and MB4 at each scan length. .

To explore regional and network-level patterns of reliability, for each trimmed duration we computed ΔICC (MB4−SB) for every ROI–ROI connection. The resulting ΔICC matrices were reordered according to large-scale functional network affiliation and hemisphere, following the widely used cortical network taxonomy of Yeo and colleagues [Yeo et al., 2011]. These connection-wise differences are presented as heatmaps, where visually distinct network blocks indicate whether MB4 reliability advantages arise within specific systems or are more globally distributed.

In addition, ROI-wise mean ΔICC values were computed by averaging each region's edge-wise differences across all of its connections. These values are displayed as horizontal bar plots adjacent to the heatmaps, reflecting the overall gain (or loss) in ICC reliability for each ROI under MB4 relative to SB.

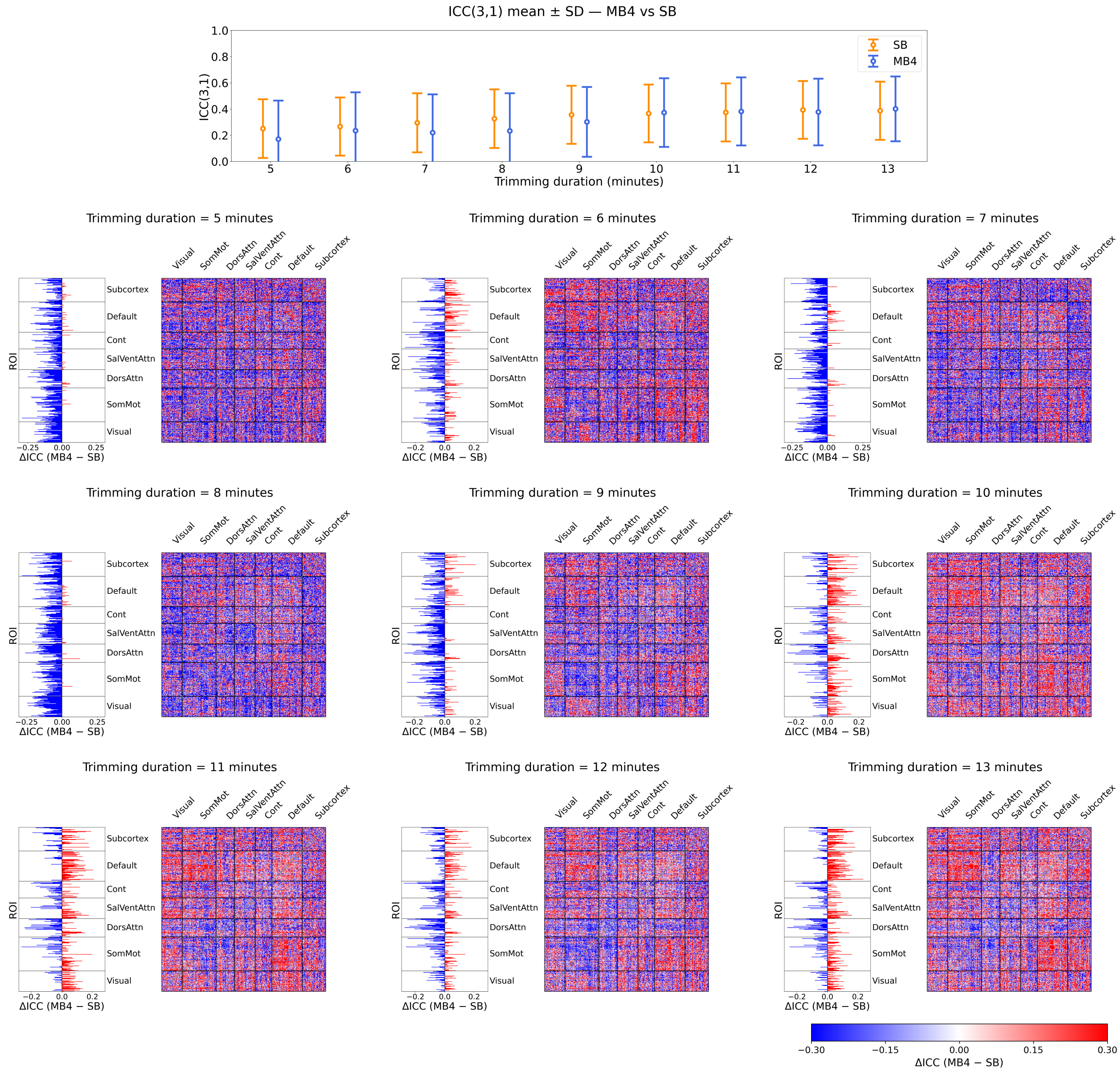

Figure S1: Edge-wise test–retest reliability benchmarking using ICC(3,1) for multiband (MB4) and single-band (SB) acquisitions across trimmed scan durations (5–13 minutes). The top plot displays global mean ICC values with standard deviation error bars for SB and MB4. The remaining panels show, for each duration, paired visualizations of connection-level ΔICC (MB4−SB) presented as reordered heatmaps, and region-wise mean ΔICC values displayed as horizontal bar plots. ROIs were arranged by functional network and hemisphere, revealing spatial patterns of reliability differences. Positive ΔICC values (red color) indicate higher stability under MB4 relative to SB; negative values (blue color) indicate the opposite.

3 Model Diagnostics

Model assumptions were evaluated using visual inspection of residual and random-effects distributions, as well as residual-versus-fitted relationships. As a complementary numerical summary, correlations between absolute residuals and fitted values were examined.

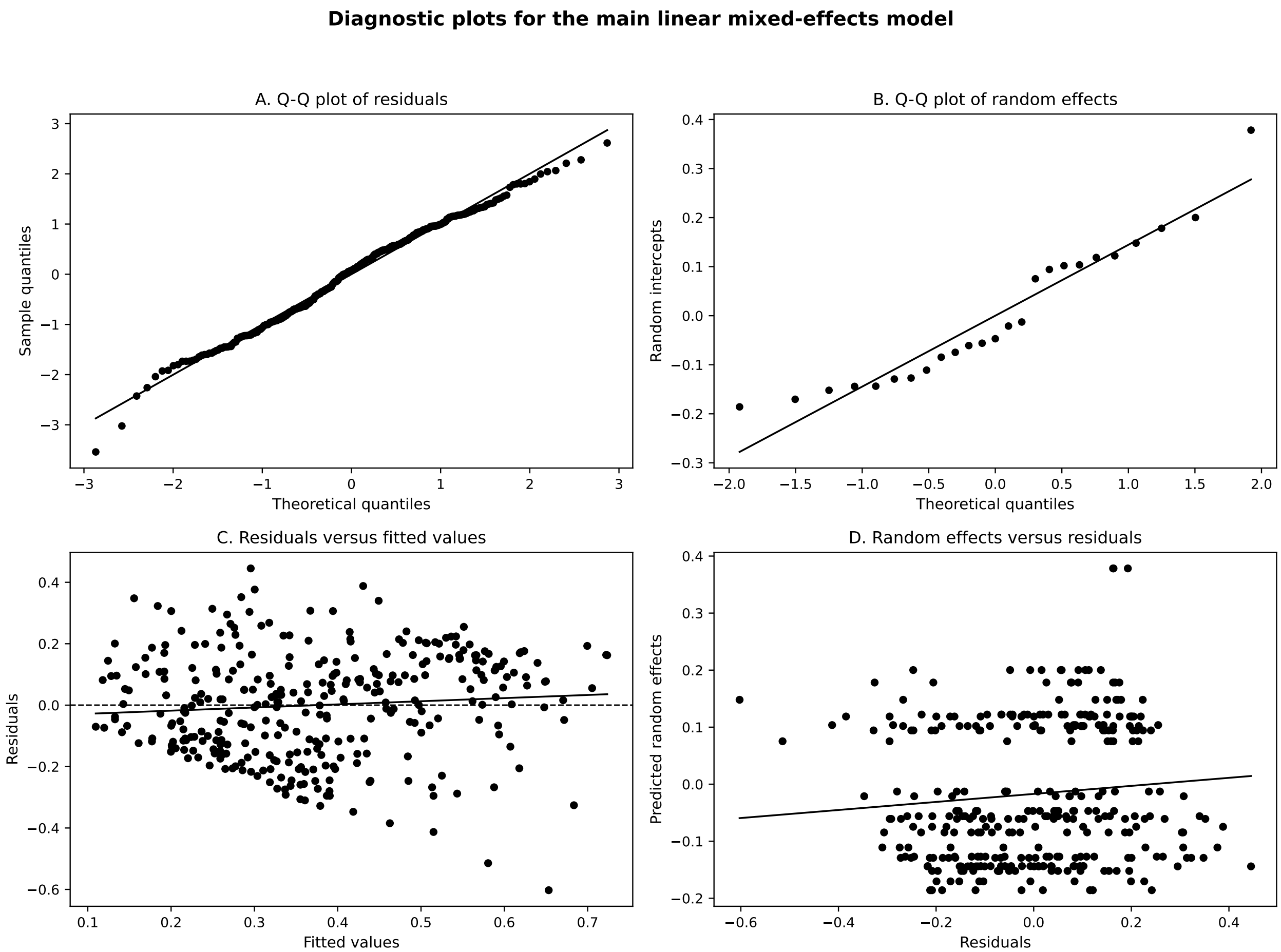

Figure S2: Diagnostic plots for the main linear mixed-effects model fitted to the primary analysis variant (Schaefer 400 + Tian S4, FD threshold = 0.4 mm, edge threshold = 20%). Panel A shows the Q–Q plot of model residuals, panel B the Q–Q plot of subject-specific random intercepts, panel C the residuals-versus-fitted-values plot, and panel D the relationship between residuals and predicted random effects.

The Q–Q plots did not indicate substantial deviations from normality, and the residuals-versus-fitted plot showed no pronounced systematic structure. The correlation between absolute residuals and fitted values was small and non-significant (Pearson $r = 0.085$, $p = 0.119$), providing no indication of pronounced heteroscedasticity. The association between residuals and predicted random effects was likewise weak (Pearson $r = 0.099$, $p = 0.069$), suggesting no substantial dependence between residuals and subject-specific random intercepts. Overall, these diagnostics did not reveal violations that would materially affect inference.

4 Power Analysis

Statistical power was evaluated using simulation-based procedures matched to the inferential framework used in the primary analyses. Data were generated under the fitted linear mixed-effects models, preserving the fixed-effects structure, contrast coding, random-intercept specification, and estimated variance components. For each scenario, 5000 simulated datasets were generated, and power was defined as the proportion of significant tests at $\alpha = 0.05$ based on Type II ANOVA.

Two primary inferential targets were considered. First, for the between-method comparison, power was evaluated in the model

$$\text{Divergence} \sim \text{Method} + \text{Trimmed} + (1 \mid \text{Subject}),$$

as a function of the absolute effect size $|\beta|$ for the contrast MB4 vs. SB. The absolute magnitude was used because the global F-test for *Method* is insensitive to effect direction.

The observed effect was $\beta = -0.1266$ (MB4 relative to SB), corresponding to $|\beta| = 0.1266$, with an estimated power of 1.000. The minimal detectable effect (MDE) for 80% power was 0.07.

Second, for the global effect of scan duration within MB4, power was evaluated in the model

$$\text{Divergence} \sim \text{Trimmed} + (1 \mid \text{Subject}),$$

treating *Trimmed* as a multi-level factor. The alternative hypothesis was parameterized using the estimated coefficient vector $\hat{\boldsymbol{\beta}}$ from the empirical MB4-only model, such that

$$\boldsymbol{\beta}(k) = k\hat{\boldsymbol{\beta}}, \quad k \geq 0,$$

which preserves the observed multilevel structure of the duration effect while varying its magnitude.

The observed effect corresponds to $k = 1$ and yielded an estimated power of 0.983. The minimal detectable effect for 80% power corresponded to $k = 0.75$, indicating that a proportional reduction of the observed effect pattern to 75% of its magnitude would remain detectable under the assumed model. This interpretation is restricted to alternatives proportional to the observed coefficient structure and does not generalize to arbitrary configurations of the *Trimmed* effect.

5 Stability Analyses under Parcellation Scheme and Data-Quality Constraints

We conducted complementary analyses to assess the robustness of our findings with respect to different parcellation schemes and data-quality constraints. Specifically, the full analytical workflow was repeated using alternative functional atlas resolutions (Schaefer 100 and 200, in addition to the primary Schaefer 400 combined with the Tian atlas) and, separately, using cortical-only ROIs. Furthermore, to evaluate the influence of motion-related preprocessing choices, all configurations were examined under two censoring thresholds (FD = 0.4 mm and FD = 1.0 mm).

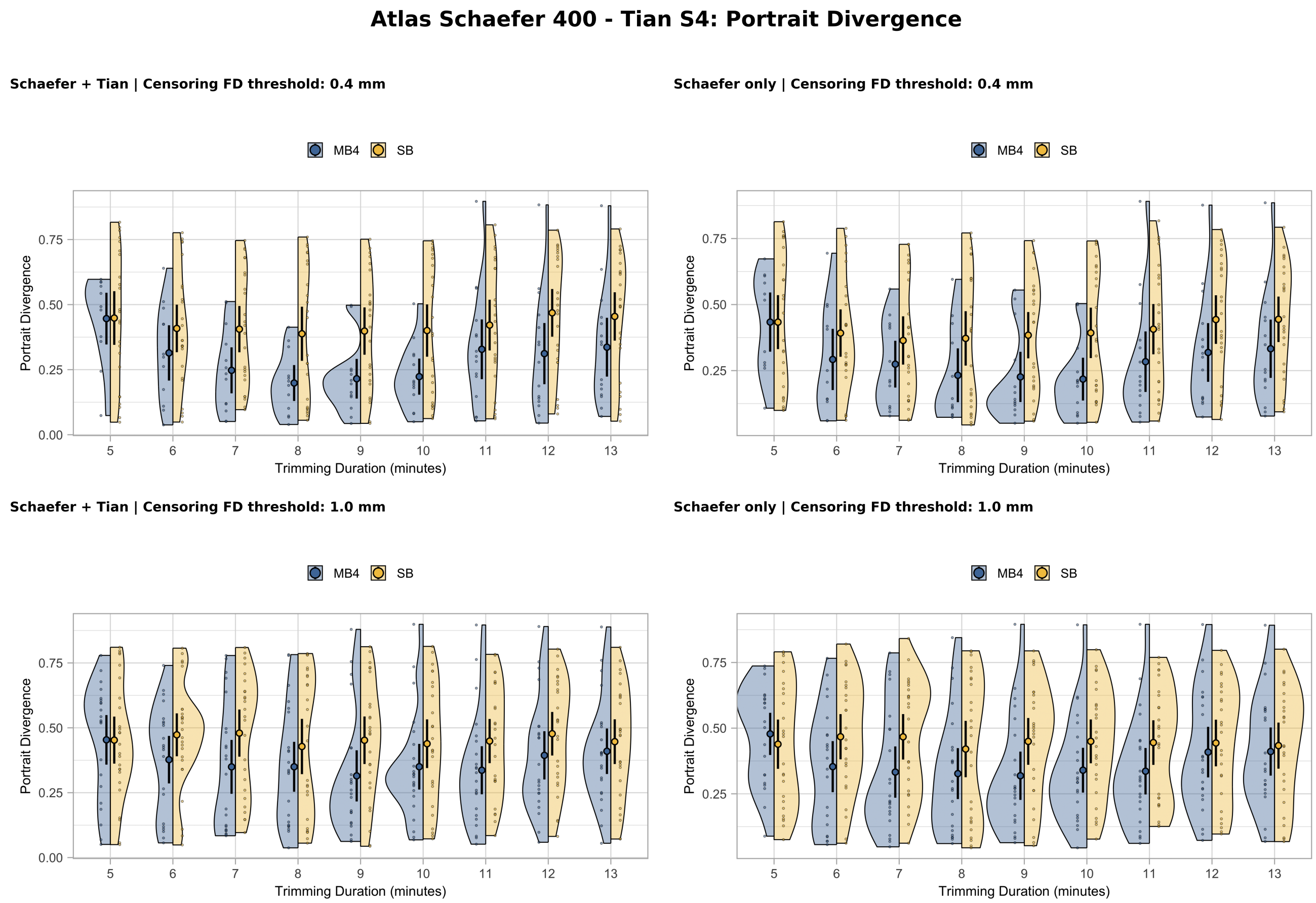

Figure S3: Split violin plot depicting the distribution of Portrait Divergence values across different trimmed scan durations (in minutes) for multiband factor 4 (MB4) and single-band (SB) acquisition methods. Each subplot represents a unique combination of brain regions (Schaefer-only vs. Schaefer + Tian) and censoring thresholds based on framewise displacement (FD = 0.4 mm vs. FD = 1.0 mm). The shapes of violins reflect kernel density estimates for each method separately, accompanied by jittered points to visualize individual data points. Black point-range markers within each violin depict mean divergence values with corresponding 95% confidence intervals.

Schaefer + Tian | Censoring FD threshold: 0.4 mm

Analysis	Result
**MB4 vs SB:**	
Mean difference:	−0.128, 95% CI: -0.172, -0.083
	ANOVA P = 2.0e-07 ***
**Effect of scan length**	
- within SB:	ANOVA P = 1.000
- within MB4:	ANOVA P = 0.002 **
**Quadratic model fit**	
- SB: optimum at	8.45 min $R^2_{adj} = 0.692$, P = 0.036 *
- MB4: optimum at	9.20 min $R^2_{adj} = 0.659$, P = 0.049 *
**Equations of fitted quadratic curves**	
- SB:	$y = 0.0037t^2 - 0.0628t + 0.6608$
- MB4:	$y = 0.0111t^2 - 0.2033t + 1.1519$

Schaefer + Tian | Censoring FD threshold: 1.0 mm

Analysis	Result
**MB4 vs SB:**	
Mean difference:	−0.086, 95% CI: -0.123, -0.05
	ANOVA P = 2.2e-05 ***
**Effect of scan length**	
- within SB:	ANOVA P = 1.000
- within MB4:	ANOVA P = 0.490
**Quadratic model fit**	
- SB: optimum at	9.76 min $R^2_{adj} = -0.471$, P = 1.000
- MB4: optimum at	9.20 min $R^2_{adj} = 0.815$, P = 0.008 **
**Equations of fitted quadratic curves**	
- SB:	$y = 0.0007t^2 - 0.0132t + 0.5149$
- MB4:	$y = 0.0064t^2 - 0.1173t + 0.8672$

Schaefer only | Censoring FD threshold: 0.4 mm

Analysis	Result
**MB4 vs SB:**	
Mean difference:	−0.116, 95% CI: -0.162, -0.071
	ANOVA P = 3.8e-06 ***
**Effect of scan length**	
- within SB:	ANOVA P = 1.000
- within MB4:	ANOVA P = 0.048 *
**Quadratic model fit**	
- SB: optimum at	8.35 min $R^2_{adj} = 0.741$, P = 0.021 *
- MB4: optimum at	9.27 min $R^2_{adj} = 0.795$, P = 0.011 *
**Equations of fitted quadratic curves**	
- SB:	$y = 0.0039t^2 - 0.0646t + 0.6457$
- MB4:	$y = 0.0099t^2 - 0.1826t + 1.0704$

Schaefer only | Censoring FD threshold: 1.0 mm

Analysis	Result
**MB4 vs SB:**	
Mean difference:	−0.080, 95% CI: -0.115, -0.045
	ANOVA P = 4.3e-05 ***
**Effect of scan length**	
- within SB:	ANOVA P = 1.000
- within MB4:	ANOVA P = 0.008 **
**Quadratic model fit**	
- SB: optimum at	5.99 min $R^2_{adj} = -0.402$, P = 1.000
- MB4: optimum at	9.09 min $R^2_{adj} = 0.696$, P = 0.034 *
**Equations of fitted quadratic curves**	
- SB:	$y = -0.0003t^2 + 0.0036t + 0.4401$
- MB4:	$y = 0.0078t^2 - 0.1410t + 0.9564$

Table S1: Summary of statistical analyses: comparison between MB4 and SB methods, and quadratic fits across scan lengths corresponding to Fig. S3 (Schaefer 400 - Tian S4 atlas). All p-values were corrected for multiple comparisons using the Bonferroni applied within each analytical specification (defined by atlas, ROI definition, and FD threshold). Significance levels after correction: * $p < 0.05$, ** $p < 0.01$, *** $p < 0.001$.

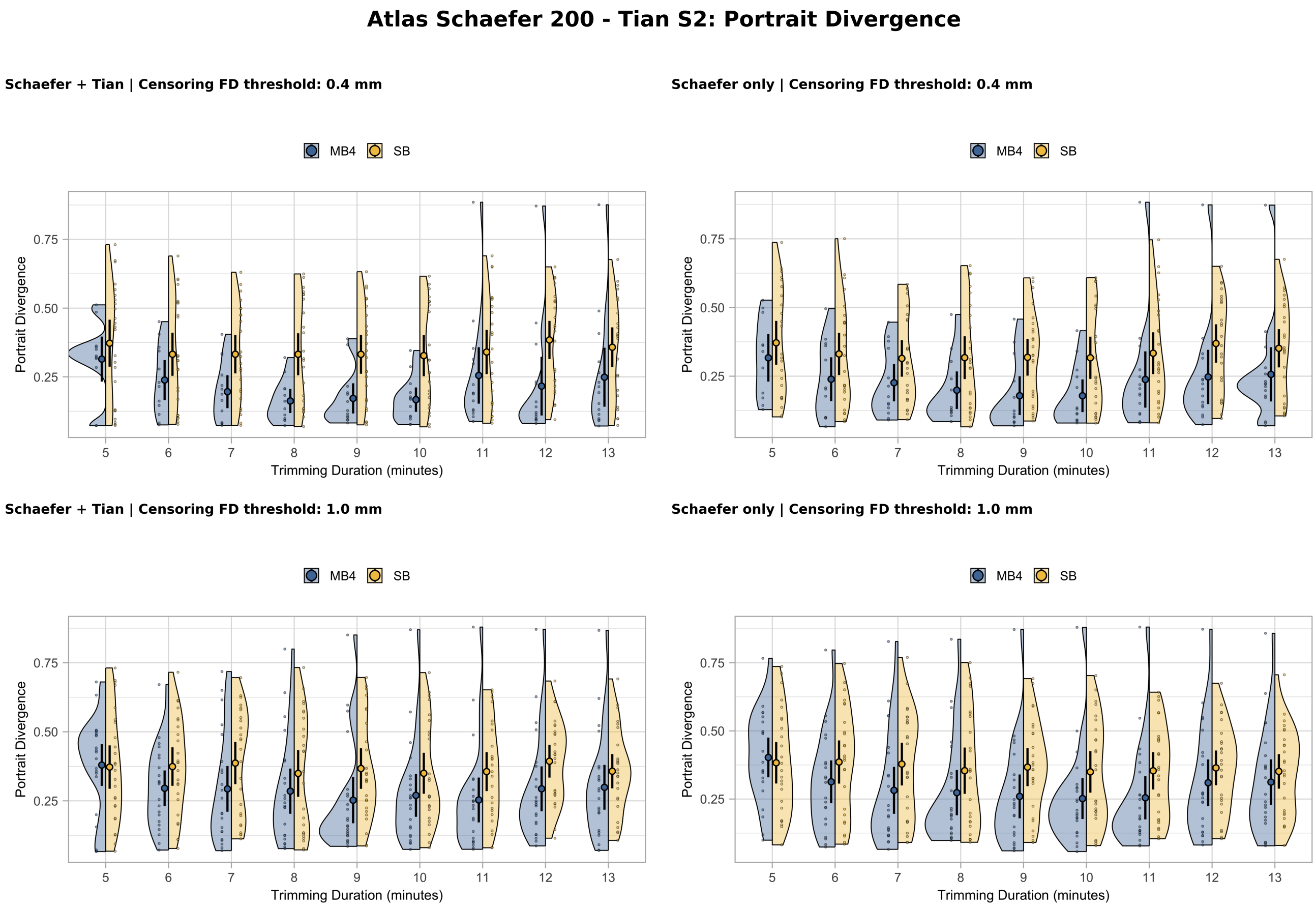

Figure S4: Split violin plot depicting the distribution of Portrait Divergence values across different trimmed scan durations (in minutes) for multiband factor 4 (MB4) and single-band (SB) acquisition methods. Each subplot represents a unique combination of brain regions (Schaefer-only vs. Schaefer + Tian) and censoring thresholds based on framewise displacement (FD = 0.4 mm vs. FD = 1.0 mm). The shapes of violins reflect kernel density estimates for each method separately, accompanied by jittered points to visualize individual data points. Black point-range markers within each violin depict mean divergence values with corresponding 95% confidence intervals.

Schaefer + Tian | Censoring FD threshold: 0.4 mm

Analysis	Result
**MB4 vs SB:**	
Mean difference:	−0.130, 95% CI: -0.164, -0.096<br>ANOVA P = 2.6e-12 ***
**Effect of scan length**	
- within SB:	ANOVA P = 1.000
- within MB4:	ANOVA P = 0.007 **
**Quadratic model fit**	
- SB: optimum at	8.63 min $R^2_{adj} = 0.324$, P = 0.377
- MB4: optimum at	9.25 min $R^2_{adj} = 0.585$, P = 0.087
**Equations of fitted quadratic curves**	
- SB:	$y = 0.0024t^2 - 0.0417t + 0.5089$
- MB4:	$y = 0.0068t^2 - 0.1264t + 0.7578$

Schaefer only | Censoring FD threshold: 0.4 mm

Analysis	Result
**MB4 vs SB:**	
Mean difference:	−0.101, 95% CI: -0.135, -0.067<br>ANOVA P = 5.3e-08 ***
**Effect of scan length**	
- within SB:	ANOVA P = 1.000
- within MB4:	ANOVA P = 0.023 *
**Quadratic model fit**	
- SB: optimum at	8.80 min $R^2_{adj} = 0.543$, P = 0.117
- MB4: optimum at	9.28 min $R^2_{adj} = 0.762$, P = 0.016 *
**Equations of fitted quadratic curves**	
- SB:	$y = 0.0030t^2 - 0.0534t + 0.5507$
- MB4:	$y = 0.0062t^2 - 0.1156t + 0.7256$

Schaefer + Tian | Censoring FD threshold: 1.0 mm

Analysis	Result
**MB4 vs SB:**	
Mean difference:	−0.079, 95% CI: -0.109, -0.05<br>ANOVA P = 9.7e-07 ***
**Effect of scan length**	
- within SB:	ANOVA P = 1.000
- within MB4:	ANOVA P = 0.056
**Quadratic model fit**	
- SB: optimum at	9.77 min $R^2_{adj} = -0.431$, P = 1.000
- MB4: optimum at	9.76 min $R^2_{adj} = 0.751$, P = 0.019 *
**Equations of fitted quadratic curves**	
- SB:	$y = 0.0007t^2 - 0.0134t + 0.4277$
- MB4:	$y = 0.0046t^2 - 0.0903t + 0.6987$

Schaefer only | Censoring FD threshold: 1.0 mm

Analysis	Result
**MB4 vs SB:**	
Mean difference:	−0.071, 95% CI: -0.1, -0.042<br>ANOVA P = 1.2e-05 ***
**Effect of scan length**	
- within SB:	ANOVA P = 1.000
- within MB4:	ANOVA P = 1.4e-04 ***
**Quadratic model fit**	
- SB: optimum at	11.88 min $R^2_{adj} = 0.546$, P = 0.114
- MB4: optimum at	9.58 min $R^2_{adj} = 0.853$, P = 0.004 **
**Equations of fitted quadratic curves**	
- SB:	$y = 0.0007t^2 - 0.0168t + 0.4546$
- MB4:	$y = 0.0065t^2 - 0.1236t + 0.8422$

Table S2: Summary of statistical analyses: comparison between MB4 and SB methods, and quadratic fits across scan lengths corresponding to Fig. S4 (Schaefer 200 - Tian S2 atlas). All p-values were corrected for multiple comparisons using the Bonferroni applied within each analytical specification (defined by atlas, ROI definition, and FD threshold). Significance levels after correction: * $p < 0.05$, ** $p < 0.01$, *** $p < 0.001$.

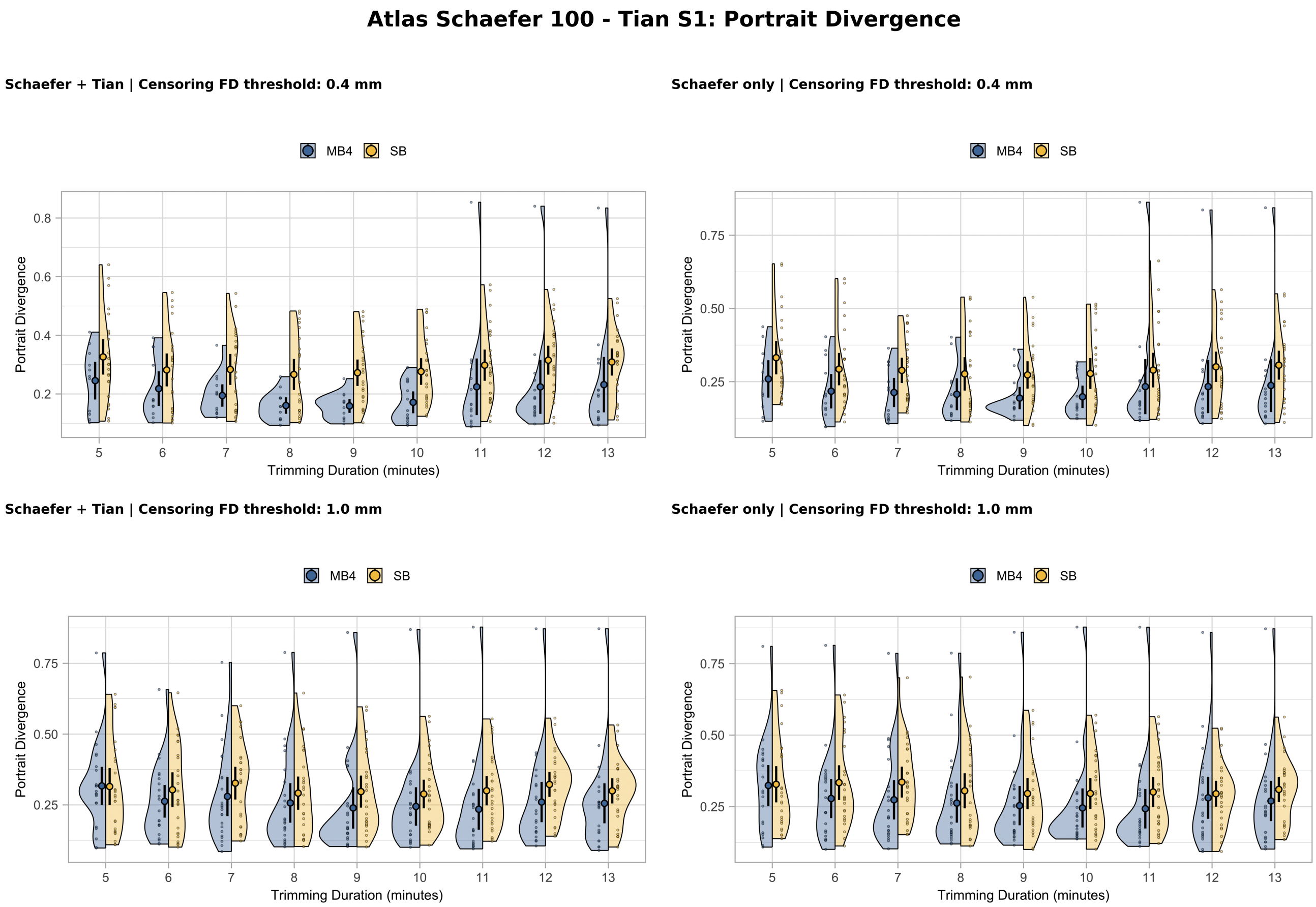

Figure S5: Split violin plot depicting the distribution of Portrait Divergence values across different trimmed scan durations (in minutes) for multiband factor 4 (MB4) and single-band (SB) acquisition methods. Each subplot represents a unique combination of brain regions (Schaefer-only vs. Schaefer + Tian) and censoring thresholds based on framewise displacement (FD = 0.4 mm vs. FD = 1.0 mm). The shapes of violins reflect kernel density estimates for each method separately, accompanied by jittered points to visualize individual data points. Black point-range markers within each violin depict mean divergence values with corresponding 95% confidence intervals.

Schaefer + Tian | Censoring FD threshold: 0.4 mm

Analysis	Result
**MB4 vs SB:**	
Mean difference:	−0.095, 95% CI: -0.118, -0.071<br>ANOVA P = 1.4e-13 ***
**Effect of scan length**	
- within SB:	ANOVA P = 1.000
- within MB4:	ANOVA P = 0.074
**Quadratic model fit**	
- SB: optimum at	8.80 min $R^2_{adj} = 0.586$, P = 0.086
- MB4: optimum at	8.94 min $R^2_{adj} = 0.640$, P = 0.057
**Equations of fitted quadratic curves**	
- SB:	$y = 0.0029t^2 - 0.0502t + 0.4938$
- MB4:	$y = 0.0046t^2 - 0.0828t + 0.5426$

Schaefer only | Censoring FD threshold: 0.4 mm

Analysis	Result
**MB4 vs SB:**	
Mean difference:	−0.067, 95% CI: -0.09, -0.043<br>ANOVA P = 2.2e-07 ***
**Effect of scan length**	
- within SB:	ANOVA P = 1.000
- within MB4:	ANOVA P = 1.000
**Quadratic model fit**	
- SB: optimum at	9.23 min $R^2_{adj} = 0.820$, P = 0.007 **
- MB4: optimum at	9.03 min $R^2_{adj} = 0.602$, P = 0.077
**Equations of fitted quadratic curves**	
- SB:	$y = 0.0027t^2 - 0.0502t + 0.5065$
- MB4:	$y = 0.0029t^2 - 0.0530t + 0.4411$

Schaefer + Tian | Censoring FD threshold: 1.0 mm

Analysis	Result
**MB4 vs SB:**	
Mean difference:	−0.045, 95% CI: -0.068, -0.021<br>ANOVA P = 1.0e-03 ***
**Effect of scan length**	
- within SB:	ANOVA P = 1.000
- within MB4:	ANOVA P = 0.090
**Quadratic model fit**	
- SB: optimum at	9.62 min $R^2_{adj} = -0.296$, P = 1.000
- MB4: optimum at	10.21 min $R^2_{adj} = 0.676$, P = 0.041 *
**Equations of fitted quadratic curves**	
- SB:	$y = 0.0008t^2 - 0.0159t + 0.3756$
- MB4:	$y = 0.0025t^2 - 0.0501t + 0.4973$

Schaefer only | Censoring FD threshold: 1.0 mm

Analysis	Result
**MB4 vs SB:**	
Mean difference:	−0.038, 95% CI: -0.062, -0.014<br>ANOVA P = 0.009 **
**Effect of scan length**	
- within SB:	ANOVA P = 1.000
- within MB4:	ANOVA P = 0.021 *
**Quadratic model fit**	
- SB: optimum at	10.98 min $R^2_{adj} = 0.512$, P = 0.142
- MB4: optimum at	9.82 min $R^2_{adj} = 0.743$, P = 0.021 *
**Equations of fitted quadratic curves**	
- SB:	$y = 0.0011t^2 - 0.0246t + 0.4347$
- MB4:	$y = 0.0029t^2 - 0.0574t + 0.5305$

Table S3: Summary of statistical analyses: comparison between MB4 and SB methods, and quadratic fits across scan lengths corresponding to Fig. S5 (Schaefer 100 - Tian S1 atlas). All p-values were corrected for multiple comparisons using the Bonferroni applied within each analytical specification (defined by atlas, ROI definition, and FD threshold). Significance levels after correction: * $p < 0.05$, ** $p < 0.01$, *** $p < 0.001$.

6 Statistical Modeling of Motion Accumulation

An important consideration in optimizing scan duration for resting-state fMRI is the progressive accumulation of motion-related artifacts over time. As shown in Figure S6, the proportion of censored volumes—in this context defined as volumes exceeding the framewise displacement threshold—increases systematically with scan duration, particularly in the MB4 acquisition. This trend indicates that longer scans are more susceptible to participant motion, which may offset the theoretical gains of higher temporal resolution. Furthermore, the variability of motion censoring between sessions increases with scan duration, suggesting reduced test–retest consistency in extended acquisitions.

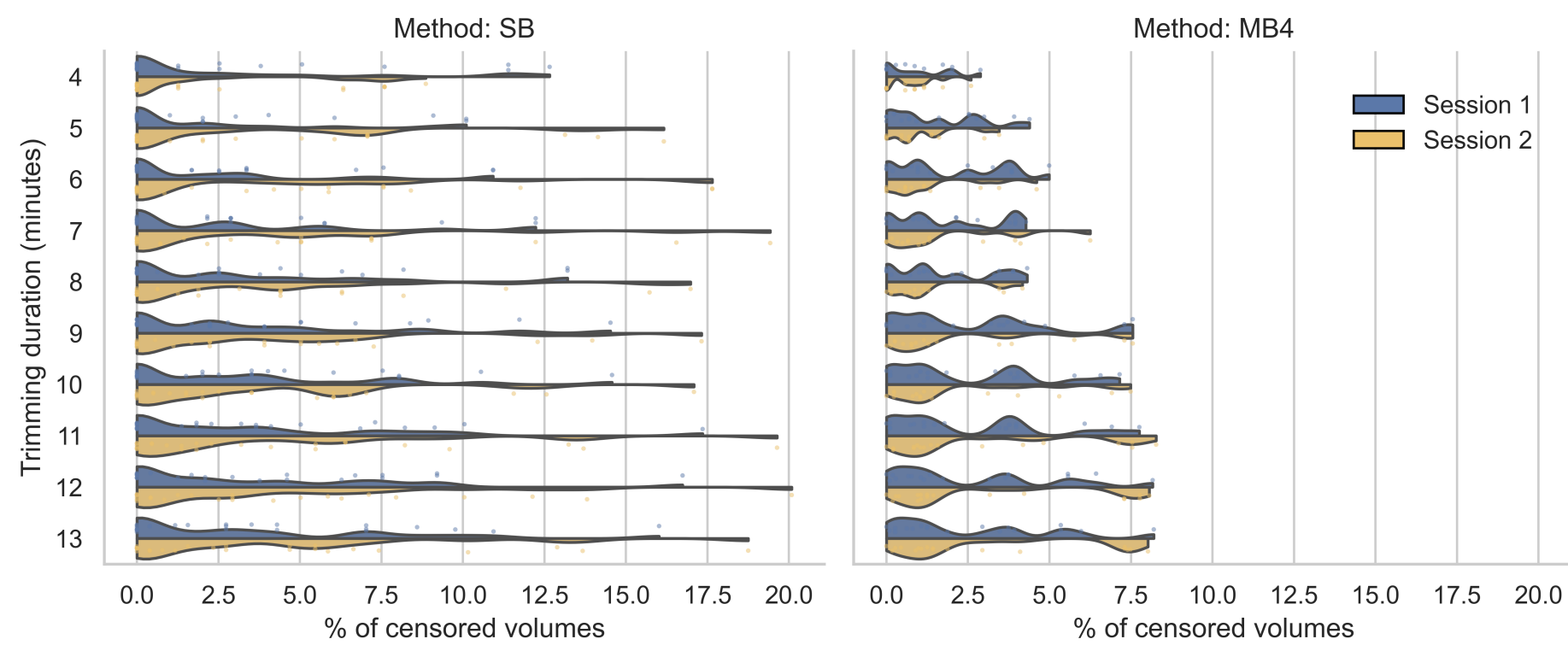

(A) Motion censoring at FD > 0.4 mm

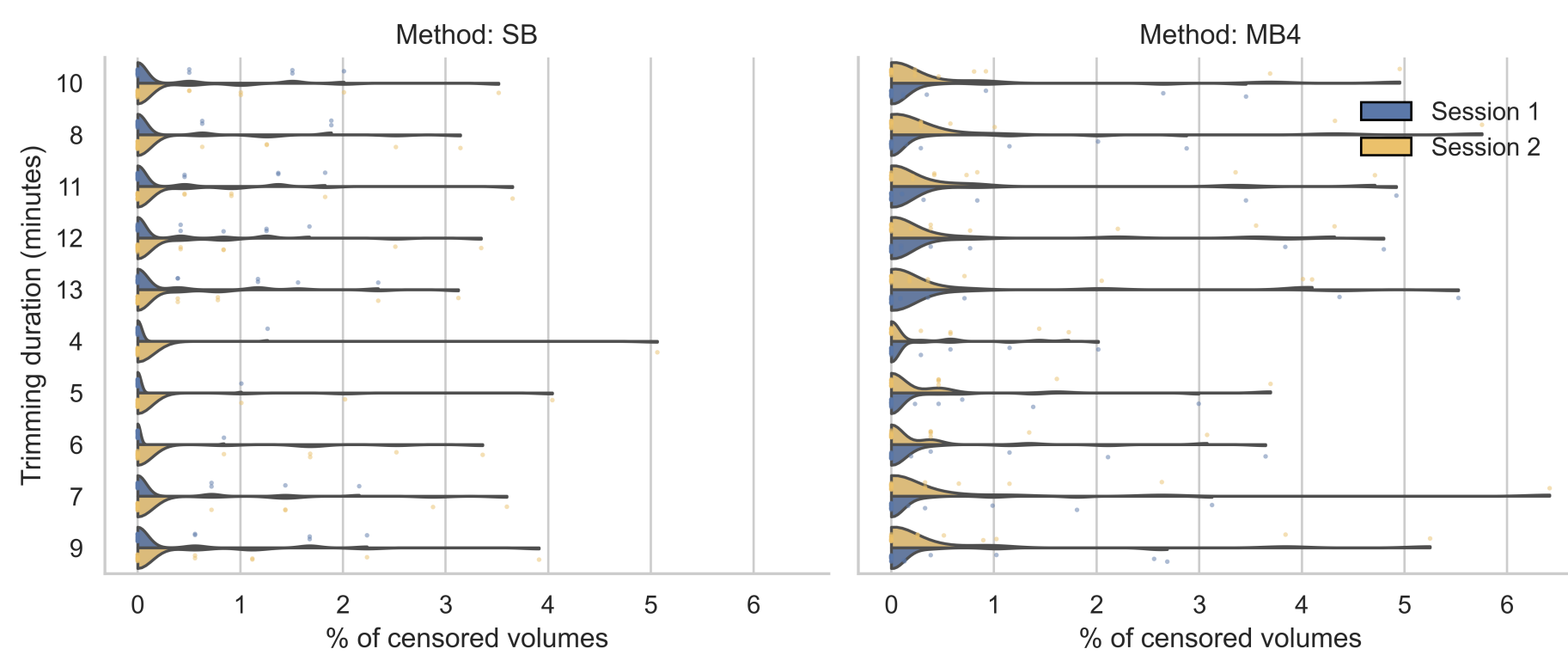

(B) Motion censoring at FD > 1.0 mm

Figure S6: Percentage of volumes censored due to excessive motion (framewise displacement, FD) across trimming durations from 5 to 13 minutes. Each panel shows split-violin plots with individual subject data points overlaid, grouped by task and session. Panel (A) uses a strict censoring threshold of 0.4 mm, while panel (B) uses a more lenient 1.0 mm.

To complement these descriptive observations, we additionally performed two statistical analyses designed to model the temporal evolution of motion artifacts and evaluate whether motion accumulation compromises longitudinal topological stability after the observed optimum at approximately 9 minutes. Both analyses were implemented in R using `lme4` [Bates et al., 2015] and `lmerTest` [Kuznetsova et al., 2017].

(1) Linear Mixed-Effects Modeling of Motion Trajectories

We first used a Linear Mixed-Effects Model (LMM) to examine how mean framewise displacement (meanFD) changes as a function of scan duration (5–13 minutes) and whether this relationship differs across sessions. The model included scan duration (minutes), session (1 vs. 2), and their interaction (minutes × session) as fixed effects, and subject as a random intercept:

$$\text{meanFD}_i = \beta_0 + \beta_1 \cdot \text{minutes}_i + \beta_2 \cdot \text{session}_i + \beta_3 \cdot (\text{minutes}_i \times \text{session}_i) + u_{\text{subject}(i)} + \varepsilon_i.$$

In the MB4 sequence, the interaction term was statistically significant ($\beta_3 = -0.0012$, $p = 0.009$), indicating that motion trajectories diverged between sessions as scan duration increased. In contrast, the interaction was non-significant in the SB acquisition ($\beta_3 = -0.0003$, $p = 0.698$).

(2) Piecewise Modeling of Motion Accumulation Around the 9-Minute Breakpoint

To explicitly test for a change in the rate of motion accumulation around the predicted optimum, we implemented a piecewise regression model with a predefined breakpoint fixed at 9 minutes:

$$\text{meanFD}_i = \beta_0 + \beta_1 \cdot t_{\text{pre},i} + \beta_2 \cdot t_{\text{post},i} + u_{\text{subject}(i)} + \varepsilon_i,$$

where $t_{\text{pre},i} = \min(t_i, 9)$ and $t_{\text{post},i} = \max(t_i - 9, 0)$. In this parametrization, β_1 represents the slope of motion accumulation before 9 minutes, whereas $\beta_1 + \beta_2$ describes the slope thereafter; thus, β_2 directly tests whether this trajectory changes beyond the 9-minute time point.

For SB data, no significant change in slope was observed ($\beta_2 = 0.0011$, $p = 0.180$), reinforcing the notion that motion artifacts do not introduce a critical time limit on SB reliability. For MB4, the change in slope was close to statistical significance ($\beta_2 = 0.0010$, $p = 0.058$).

7 MB4 Undersampling Analyses

We evaluated whether the topological stability advantage observed for full-resolution MB4 data is driven by increased temporal sampling. To this end, MB4 acquisitions were artificially downsampled to match the sampling density of SB data (denoted as MB4_ds4) by retaining every fourth volume prior to denoising, while keeping acquisition parameters otherwise unchanged. Topological stability was then re-estimated using Portrait Divergence, following the same modelling framework as in the main analysis: for each trimmed scan duration (5–13 minutes), stability was modelled as a function of acquisition method (SB, MB4, MB4_ds4) and scan length, with subject included as a random intercept. Mixed-effects models were fitted in R using the `lme4` package [Bates et al., 2015], with inference provided by `lmerTest` [Kuznetsova et al., 2017]. Pairwise contrasts were derived using Tukey-adjusted comparisons from `emmeans` [Lenth, 2023].

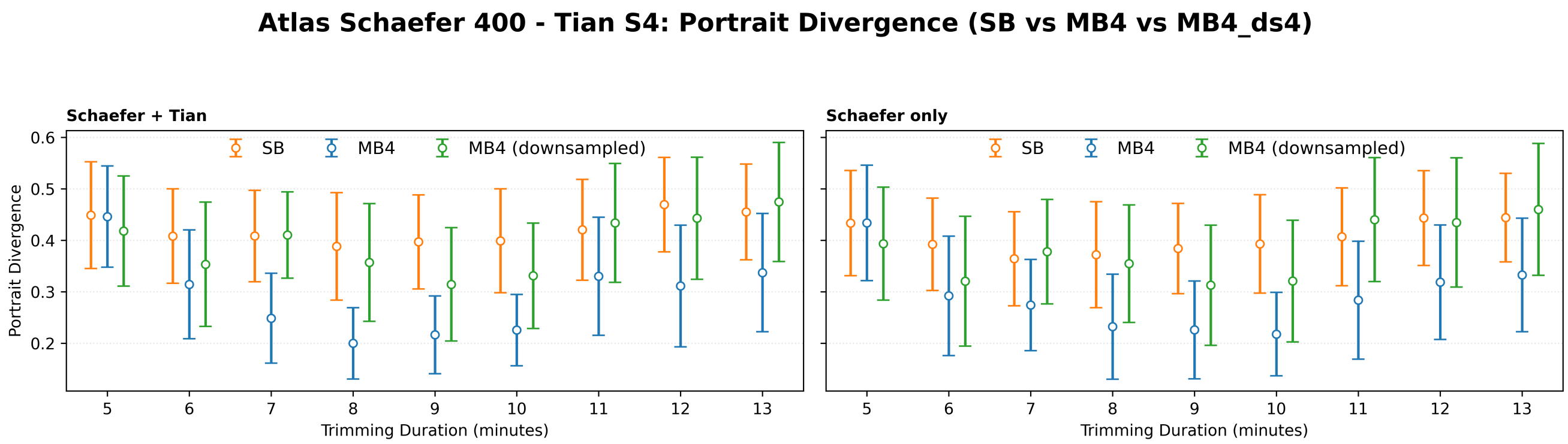

Figure S7: Error-bar plots of Portrait Divergence for single-band (SB), multiband factor 4 (MB4) and downsampled MB4 (MB4_ds4) across trimmed scan durations (5–13 minutes). The downsampled MB4 condition was constructed to match the number of volumes in the SB acquisition. Each subplot corresponds to a combination of brain regions (Schaefer + Tian vs. Schaefer only). Circles denote marginal means and vertical lines represent 95% confidence intervals.

Schaefer + Tian

**Analysis**	**Result**
**Main effect of Method**	$ANOVA\ P = 1.2 \times 10^{-7}$ ∗∗∗
**Pairwise (Tukey)**	
SB vs MB4	$\Delta = 0.125,\ P = 2.7 \times 10^{-7}$ ∗∗∗
SB vs MB4_ds4	$\Delta = 0.021,\ P = 0.630$
MB4 vs MB4_ds4	$\Delta = -0.104,\ P = 3.9 \times 10^{-5}$ ∗∗∗

Schaefer only

**Analysis**	**Result**
**Main effect of Method**	$ANOVA\ P = 1.9 \times 10^{-6}$ ∗∗∗
**Pairwise (Tukey)**	
SB vs MB4	$\Delta = 0.116,\ P = 3.0 \times 10^{-6}$ ∗∗∗
SB vs MB4_ds4	$\Delta = 0.023,\ P = 0.602$
MB4 vs MB4_ds4	$\Delta = -0.094,\ P = 3.2 \times 10^{-4}$ ∗∗∗

Table S4: Summary of mixed-effects analyses comparing SB, MB4 and MB4_ds4 acquisition schemes corresponding to Fig. S7 (Schaefer 400 - Tian S4 atlas). Reported results include the main effect of acquisition method (Method) and pairwise Tukey comparisons (SB vs MB4, SB vs MB4_ds4, MB4 vs MB4_ds4).

8 Edge Thresholding Sensitivity Analyses

To assess whether the observed effects were dependent on graph sparsity, all Portrait Divergence analyses were repeated using alternative edge retention thresholds of 10%, 15%, 25%, and 30% of the strongest positive connections, in addition to the primary 20% threshold used in the main analyses. Thresholding was applied independently for each participant and session following Fisher's z-transformation and exclusion of negative correlations, after which Portrait Divergence was recomputed identically across trimming durations.

Statistical models and inference procedures were identical to those used in the main reliability analysis. All p-values were corrected for multiple comparisons across thresholding settings using Bonferroni adjustment.

	Edge filtering threshold				
Top % edges	10	15	20	25	30
MB4 vs SB: mean diff	-0.135	-0.136	-0.128	-0.121	-0.117
(95% CI)	(-0.179, -0.090)	(-0.180, -0.091)	(-0.172, -0.083)	(-0.165, -0.077)	(-0.160, -0.073)
MB4 vs SB: ANOVA P	2.7e-08 ***	2.1e-08 ***	2.0e-07 ***	4.4e-07 ***	7.4e-07 ***
Effect of scan length					
(SB): ANOVA P	1.000	1.000	1.000	1.000	1.000
(MB4): ANOVA P	0.227	0.051	0.002 **	4.7e-05 ***	8.8e-06 ***
Quadratic optimum					
SB [min]	8.79	8.56	8.45	8.55	8.57
MB4 [min]	9.26	9.23	9.20	9.14	9.09

Table S5: Summary of statistical analyses assessing robustness to edge thresholding choices (Schaefer 400 + Tian S4, FD = 0.4 mm). For each edge retention level (10–30% of strongest positive connections), results include mean portrait divergence differences between MB4 and SB, confidence intervals, and model-based assessment of scan-length effects. Optimal durations were derived from quadratic fits. All p-values were corrected for multiple comparisons using the Bonferroni method. Significance levels after correction: * $p < 0.05$, ** $p < 0.01$, *** $p < 0.001$.

9 Sliding-Window Sensitivity Analysis

To address the potential nested dependence introduced by cumulative trimming, we performed an additional sensitivity analysis based on sliding windows of fixed length. For each subject and acquisition method, the preprocessed time series was segmented into windows of fixed duration (5–10 minutes). For each window length, windows were generated across the entire scan using a step size of 1 minute (i.e., successive windows were shifted by one minute).

Within each window, nuisance regression, band-pass filtering, and motion censoring (FD threshold) were applied identically to the main analysis. Functional connectivity matrices were then computed, and Portrait Divergence was evaluated between session 1 and session 2 for each corresponding window.

For each subject, acquisition method, and window length, the resulting set of divergence values (across all windows) was aggregated using the median, yielding a single summary measure per subject-method-length combination. This aggregation step yields a single representative estimate of between-session stability for each subject, method, and window length, thereby preserving the subject-level unit of analysis used in the primary model while summarizing variability across temporal segments of the scan.

Statistical inference was conducted using the same linear mixed-effects framework as in the primary analysis.

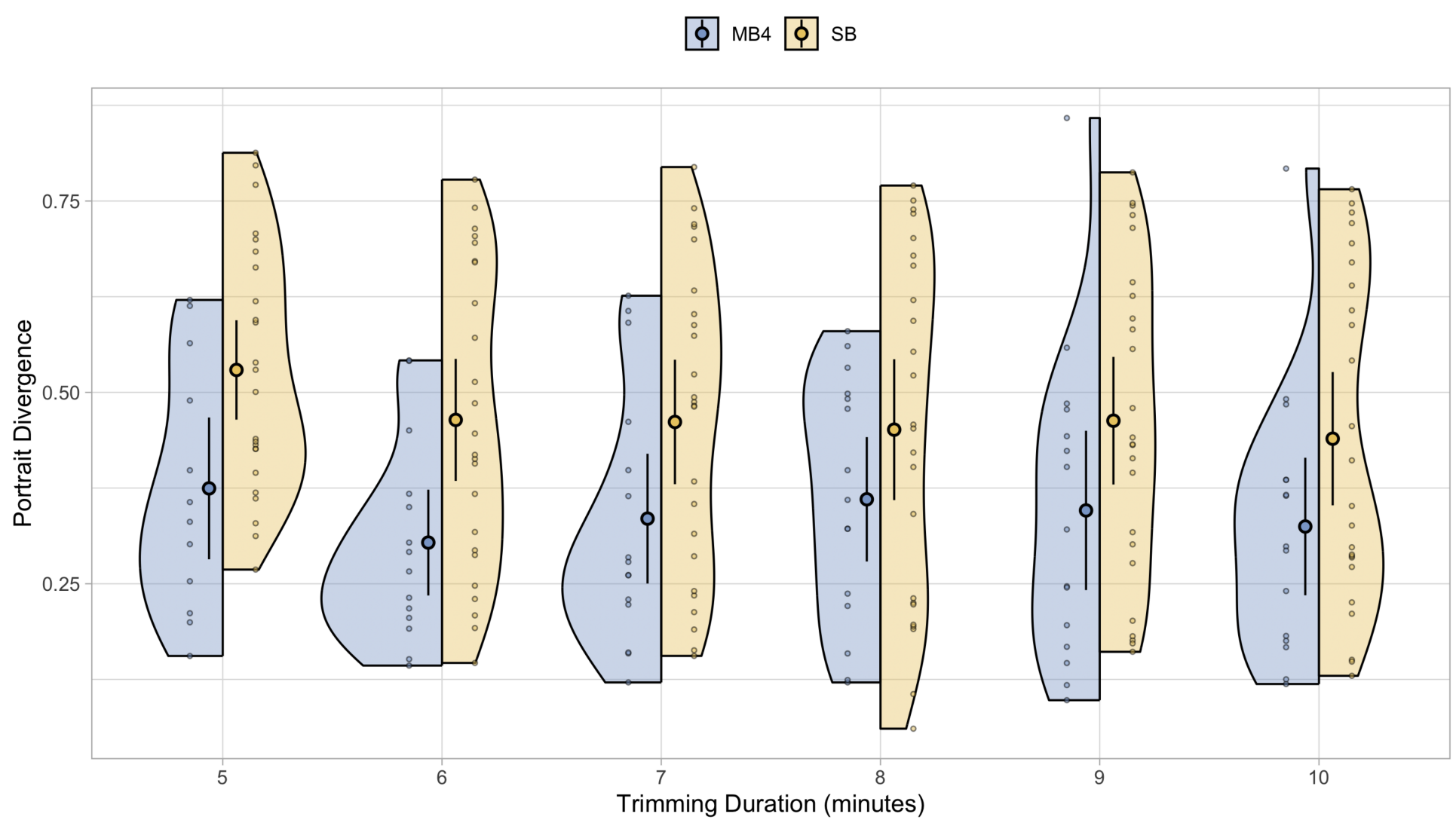

Figure S8: Sliding-window analysis of Portrait Divergence as a function of window length. Each point represents a subject-level aggregated value (median across windows), and violins illustrate the distribution across participants.

**Analysis**	**Result**
**MB4 vs SB:**	
Mean difference:	-0.113, 95% CI: -0.155, -0.07
	ANOVA $P = 2.1 \times 10^{-6}$ ***
**Effect of scan length**	
- within SB:	ANOVA $P = 0.055$
- within MB4:	ANOVA $P = 1.000$
**Quadratic model fit**	
- SB: optimum at	8.89 min, $R^2_{\text{adj}} = 0.474$, $P = 0.482$
- MB4: optimum at	8.69 min, $R^2_{\text{adj}} = -1.354$, $P = 1.000$
**Equations of fitted quadratic curves**	
- SB:	$y = 0.0048t^2 - 0.0845t + 0.8207$
- MB4:	$y = 0.0012t^2 - 0.0203t + 0.4241$

Table S6: Summary of statistical results for the sliding-window sensitivity analysis.

The sliding-window analysis yielded results consistent with the primary cumulative analysis. The MB4 acquisition

retained a significant advantage over SB, with lower Portrait Divergence across all window lengths.

As expected, no significant effect of window length was observed within either method. This attenuation of the duration effect is a direct consequence of the sliding-window design, which averages over temporal positions within the scan and therefore reduces the influence of motion accumulation that increases with elapsed scan time.

Quadratic model fits suggested an optimal window length in the range of approximately 8.7–8.9 minutes for both methods. However, these fits were not statistically significant, indicating that the precise location of the optimum cannot be reliably inferred in this analysis.

Taken together, these results indicate that the superior stability of MB4 remains robust when the deterministic nesting of cumulative segments is mitigated. At the same time, the absence of a duration effect in this analysis is consistent with its design and supports the interpretation that the approximately 9-minute optimum observed in the cumulative analysis reflects a trade-off between increasing data quantity and the accumulation of motion-related degradation over time, rather than an artifact of nested dependence.

10 Data Availability Across Trimming Durations and Motion Censoring

Minutes	Volumes		FD > 0.4 mm		FD > 1.0 mm	
	MB4	SB	MB4	SB	MB4	SB
5	434	99	11	24	21	25
6	521	119	12	24	21	25
7	608	139	13	24	22	25
8	695	159	12	24	22	25
9	781	179	13	24	22	25
10	868	199	13	24	22	25
11	955	219	15	24	22	25
12	1042	239	15	24	22	25
13	1122	256	15	24	22	25

Table S7: Number of volumes retained following trimming (MB4 and SB) and number of subjects with usable data from **both sessions**, shown for each trimming duration (minutes) and acquisition method. Values are reported separately for two motion censoring thresholds: strict (FD > 0.4 mm) and lenient (FD > 1.0 mm).

References

Alexandre Abraham, Fabian Pedregosa, Michael Eickenberg, Philippe Gervais, Andreas Mueller, Jean Kossaifi, Alexandre Gramfort, Bertrand Thirion, and Gael Varoquaux. Machine learning for neuroimaging with scikit-learn. *Frontiers in Neuroinformatics*, 8, 2014. ISSN 1662-5196. doi: 10.3389/fninf.2014.00014. URL `https://www.frontiersin.org/articles/10.3389/fninf.2014.00014/full`.

B.B. Avants, C.L. Epstein, M. Grossman, and J.C. Gee. Symmetric diffeomorphic image registration with cross-correlation: Evaluating automated labeling of elderly and neurodegenerative brain. *Medical Image Analysis*, 12 (1):26–41, 2008. ISSN 1361-8415. doi: 10.1016/j.media.2007.06.004. URL `https://www.sciencedirect.com/science/article/pii/S1361841507000606`.

Douglas Bates, Martin Mächler, Ben Bolker, and Steve Walker. Fitting linear mixed-effects models using lme4. *Journal of Statistical Software*, 67(1):1–48, 2015. doi: 10.18637/jss.v067.i01.

Yashar Behzadi, Khaled Restom, Joy Liau, and Thomas T. Liu. A component based noise correction method (CompCor) for BOLD and perfusion based fmri. *NeuroImage*, 37(1):90–101, 2007. ISSN 1053-8119. doi: 10.1016/j.neuroimage.2007.04.042. URL `https://www.sciencedirect.com/science/article/pii/S1053811907003837`.

R. Ciric, William H. Thompson, R. Lorenz, M. Goncalves, E. MacNicol, C. J. Markiewicz, Y. O. Halchenko, S. S. Ghosh, K. J. Gorgolewski, R. A. Poldrack, and O. Esteban. TemplateFlow: FAIR-sharing of multi-scale, multi-species brain models. *Nature Methods*, 19:1568–1571, 2022. doi: 10.1038/s41592-022-01681-2.

Oscar Esteban, Ross Blair, Christopher J. Markiewicz, Shoshana L. Berleant, Craig Moodie, Feilong Ma, Ayse Ilkay Isik, Asier Erramuzpe, Mathias Kent, James D. andGoncalves, Elizabeth DuPre, Kevin R. Sitek, Daniel E. P. Gomez, Daniel J. Lurie, Zhifang Ye, Russell A. Poldrack, and Krzysztof J. Gorgolewski. fmriprep 25.0.0. *Software*, 2018. doi: 10.5281/zenodo.852659.

Oscar Esteban, Christopher Markiewicz, Ross W Blair, Craig Moodie, Ayse Ilkay Isik, Asier Erramuzpe Aliaga, James Kent, Mathias Goncalves, Elizabeth DuPre, Madeleine Snyder, Hiroyuki Oya, Satrajit Ghosh, Jessey Wright, Joke Durnez, Russell Poldrack, and Krzysztof Jacek Gorgolewski. fMRIPrep: a robust preprocessing pipeline for functional MRI. *Nature Methods*, 16:111–116, 2019. doi: 10.1038/s41592-018-0235-4.

VS Fonov, AC Evans, RC McKinstry, CR Almli, and DL Collins. Unbiased nonlinear average age-appropriate brain templates from birth to adulthood. *NeuroImage*, 47, Supplement 1:S102, 2009. doi: 10.1016/S1053-8119(09)70884-5.

Matthias Gamer, Jim Lemon, Ian Fellows, and Puspendra Singh. *irr: Various Coefficients of Interrater Reliability and Agreement*, 2012. URL `https://CRAN.R-project.org/package=irr`. R package version 0.84.

K. Gorgolewski, C. D. Burns, C. Madison, D. Clark, Y. O. Halchenko, M. L. Waskom, and S. Ghosh. Nipype: a flexible, lightweight and extensible neuroimaging data processing framework in python. *Frontiers in Neuroinformatics*, 5:13, 2011. doi: 10.3389/fninf.2011.00013.

Krzysztof J. Gorgolewski, Oscar Esteban, Christopher J. Markiewicz, Erik Ziegler, David Gage Ellis, Michael Philipp Notter, Dorota Jarecka, Hans Johnson, Christopher Burns, Alexandre Manhães-Savio, Carlo Hamalainen, Benjamin Yvernault, Taylor Salo, Kesshi Jordan, Mathias Goncalves, Michael Waskom, Daniel Clark, Jason Wong, Fred Loney, Marc Modat, Blake E Dewey, Cindee Madison, Matteo Visconti di Oleggio Castello, Michael G. Clark, Michael Dayan, Dav Clark, Anisha Keshavan, Basile Pinsard, Alexandre Gramfort, Shoshana Berleant, Dylan M. Nielson, Salma Bougacha, Gael Varoquaux, Ben Cipollini, Ross Markello, Ariel Rokem, Brendan Moloney, Yaroslav O. Halchenko, Demian Wassermann, Michael Hanke, Christian Horea, Jakub Kaczmarzyk, Gilles de Hollander, Elizabeth DuPre, Ashley Gillman, David Mordom, Colin Buchanan, Rosalia Tungaraza, Wolfgang M. Pauli, Shariq Iqbal, Sharad Sikka, Matteo Mancini, Yannick Schwartz, Ian B. Malone, Mathieu Dubois, Caroline Frohlich, David Welch, Jessica Forbes, James Kent, Aimi Watanabe, Chad Cumba, Julia M. Huntenburg, Erik Kastman, B. Nolan Nichols, Arman Eshaghi, Daniel Ginsburg, Alexander Schaefer, Benjamin Acland, Steven Giavasis, Jens Kleesiek, Drew Erickson, René Küttner, Christian Haselgrove, Carlos Correa, Ali Ghayoor, Franz Liem, Jarrod Millman, Daniel Haehn, Jeff Lai, Dale Zhou, Ross Blair, Tristan Glatard, Mandy Renfro, Siqi Liu, Ari E. Kahn, Fernando Pérez-García, William Triplett, Leonie Lampe, Jörg Stadler, Xiang-Zhen Kong, Michael Hallquist, Andrey Chetverikov, John Salvatore, Anne Park, Russell Poldrack, R. Cameron Craddock, Souheil Inati, Oliver Hinds, Gavin Cooper, L. Nathan Perkins, Ana Marina,

Aaron Mattfeld, Maxime Noel, Lukas Snoek, K Matsubara, Brian Cheung, Simon Rothmei, Sebastian Urchs, Joke Durnez, Fred Mertz, Daniel Geisler, Andrew Floren, Stephan Gerhard, Paul Sharp, Miguel Molina-Romero, Alejandro Weinstein, William Broderick, Victor Saase, Sami Kristian Andberg, Robbert Harms, Kai Schlamp, Jaime Arias, Dimitri Papadopoulos Orfanos, Claire Tarbert, Arielle Tambini, Alejandro De La Vega, Thomas Nickson, Matthew Brett, Marcel Falkiewicz, Kornelius Podranski, Janosch Linkersdörfer, Guillaume Flandin, Eduard Ort, Dmitry Shachnev, Daniel McNamee, Andrew Davison, Jan Varada, Isaac Schwabacher, John Pellman, Martin Perez-Guevara, Ranjeet Khanuja, Nicolas Pannetier, Conor McDermottroe, and Satrajit Ghosh. Nipype. *Software*, 2018. doi: 10.5281/zenodo.596855.

Douglas N Greve and Bruce Fischl. Accurate and robust brain image alignment using boundary-based registration. *NeuroImage*, 48(1):63–72, 2009. ISSN 1095-9572. doi: 10.1016/j.neuroimage.2009.06.060.

Mark Jenkinson and Stephen Smith. A global optimisation method for robust affine registration of brain images. *Medical Image Analysis*, 5(2):143–156, 2001. ISSN 1361-8415. doi: 10.1016/S1361-8415(01)00036-6. URL `https://www.sciencedirect.com/science/article/pii/S1361841501000366`.

Mark Jenkinson, Peter Bannister, Michael Brady, and Stephen Smith. Improved optimization for the robust and accurate linear registration and motion correction of brain images. *NeuroImage*, 17(2):825–841, 2002. ISSN 1053-8119. doi: 10.1006/nimg.2002.1132. URL `https://www.sciencedirect.com/science/article/pii/S1053811902911328`.

Alexandra Kuznetsova, Per B. Brockhoff, and Rune H. B. Christensen. lmertest package: Tests in linear mixed effects models. *Journal of Statistical Software*, 82(13):1–26, 2017. doi: 10.18637/jss.v082.i13.

Russell V. Lenth. emmeans: Estimated marginal means, aka least-squares means. 2023. URL `https://CRAN.R-project.org/package=emmeans`. R package version 1.8.9.

Rémi Patriat, Richard C. Reynolds, and Rasmus M. Birn. An improved model of motion-related signal changes in fMRI. *NeuroImage*, 144, Part A:74–82, January 2017. ISSN 1053-8119. doi: 10.1016/j.neuroimage.2016.08.051. URL `https://www.sciencedirect.com/science/article/pii/S1053811916304360`.

Jonathan D. Power, Anish Mitra, Timothy O. Laumann, Abraham Z. Snyder, Bradley L. Schlaggar, and Steven E. Petersen. Methods to detect, characterize, and remove motion artifact in resting state fmri. *NeuroImage*, 84 (Supplement C):320–341, 2014. ISSN 1053-8119. doi: 10.1016/j.neuroimage.2013.08.048. URL `https://www.sciencedirect.com/science/article/pii/S1053811913009117`.

Martin Reuter, Herminia Diana Rosas, and Bruce Fischl. Highly accurate inverse consistent registration: A robust approach. *NeuroImage*, 53(4):1181–1196, 2010. doi: 10.1016/j.neuroimage.2010.07.020.

Theodore D. Satterthwaite, Mark A. Elliott, Raphael T. Gerraty, Kosha Ruparel, James Loughead, Monica E. Calkins, Simon B. Eickhoff, Hakon Hakonarson, Ruben C. Gur, Raquel E. Gur, and Daniel H. Wolf. An improved framework for confound regression and filtering for control of motion artifact in the preprocessing of resting-state functional connectivity data. *NeuroImage*, 64(1):240–256, 2013. ISSN 10538119. doi: 10.1016/j.neuroimage.2012.08.052. URL `https://www.sciencedirect.com/science/article/pii/S1053811912008609`.

N. J. Tustison, B. B. Avants, P. A. Cook, Y. Zheng, A. Egan, P. A. Yushkevich, and J. C. Gee. N4itk: Improved n3 bias correction. *IEEE Transactions on Medical Imaging*, 29(6):1310–1320, 2010. ISSN 0278-0062. doi: 10.1109/TMI.2010.2046908.

B. T. Thomas Yeo, Fenna M. Krienen, Jorge Sepulcre, Mert R. Sabuncu, Danial Lashkari, Marisa Hollinshead, Joshua L. Roffman, Jordan W. Smoller, László Zöllei, Jonathan R. Polimeni, et al. The organization of the human cerebral cortex estimated by intrinsic functional connectivity. *Journal of Neurophysiology*, 106(3):1125–1165, 2011.

Y. Zhang, M. Brady, and S. Smith. Segmentation of brain MR images through a hidden markov random field model and the expectation-maximization algorithm. *IEEE Transactions on Medical Imaging*, 20(1):45–57, 2001. ISSN 0278-0062. doi: 10.1109/42.906424.
